# Reach out behavioral intervention for hypertension initiated in the emergency department connecting multiple health systems: study protocol for a randomized control trial

**DOI:** 10.1186/s13063-020-04340-z

**Published:** 2020-06-03

**Authors:** William J. Meurer, Mackenzie Dinh, Kelley M. Kidwell, Adam Flood, Emily Champoux, Candace Whitfield, Deborah Trimble, Joan Cowdery, Dominic Borgialli, Sacha Montas, Rebecca Cunningham, Lorraine R. Buis, Devin Brown, Lesli Skolarus

**Affiliations:** 1 Department of Emergency Medicine, University of Michigan, Ann Arbor, MI USA; 2 Department of Neurology, University of Michigan, Ann Arbor, MI USA; 3 Stroke Program, University of Michigan, Ann Arbor, MI USA; 4 Institute for Healthcare Policy and Innovation, University of Michigan, Ann Arbor, MI USA; 5 Michigan Institute for Integrative Research in Critical Care (MCIRCC), University of Michigan, Ann Arbor, MI USA; 6 Department of Biostatistics, School of Public Health, University of Michigan, Ann Arbor, MI USA; 7 School of Health Promotion and Human Performance, Eastern Michigan University, Ypsilanti, MI USA; 8 Department of Emergency Medicine, Hurley Medical Center, Flint, MI USA; 9 Department of Family Medicine, University of Michigan, Ann Arbor, MI USA

**Keywords:** Hypertension, Emergency medicine, Randomized clinical trial, Randomized controlled trial, Multiphase optimization strategy

## Abstract

**Background:**

Hypertension is the most important modifiable risk factor for cardiovascular disease, the leading cause of mortality in the United States. The Emergency Department represents an underutilized opportunity to impact difficult-to-reach populations. There are 136 million visits to the Emergency Department each year and nearly all have at least one blood pressure measured and recorded. Additionally, an increasing number of African Americans and socioeconomically disadvantaged patients are overrepresented in the Emergency Department patient population. In the age of electronic health records and mobile health, the Emergency Department has the potential to become an integral partner in chronic disease management. The electronic health records in conjunction with mobile health behavior interventions can be leveraged to identify hypertensive patients to impact otherwise unreached populations.

**Methods:**

Reach Out is a factorial trial studying multicomponent, behavioral interventions to reduce blood pressure in the Emergency Department patient population. Potential participants are identified by automated alerts from the electronic health record and, following consent, receive a blood pressure cuff to take home. During the initial screening phase, they are prompted to submit weekly blood pressure readings. Responders with persistent hypertension are then randomized into one of three component arms, consisting of varying intensity levels: (1) healthy behavior text messaging (daily vs. none), (2) blood pressure self-monitoring (daily vs. weekly), and (3) facilitated primary care provider appointment scheduling and transportation (yes vs. no). If participants are randomized to receive facilitated primary care provider appointment scheduling and are not established with a primary care provider, care will be established at a local Federally Qualified Health Center. Participants are followed for 12 months.

**Discussion:**

The Reach Out study is designed to determine which behavioral intervention components or ‘dose’ of components contributes to a reduction in systolic blood pressure after 1 year (Aim 1). The study will also assess the effect of primary care provider appointment assistance on total primary care follow-up visits of hypertensive patients treated in an urban, safety net Emergency Department (Aim 2). Ideally, the Reach Out system will contribute to hypertension management, serving as a model for safety net hospitals and Federally Qualified Health Centers to improve chronic disease management in underserved communities.

**Trial registration:**

This study was registered at clinicaltrials.gov, identifier NCT03422718. The record was first available to the public on January 30, 2018 prior to the enrollment of patients on March 25, 2019.

Note: the numbers in curly brackets in this protocol refer to SPIRIT checklist item numbers. The order of the items has been modified to group similar items (see http://www.equator-network.org/reporting-guidelines/spirit-2013-statement-defining-standard-protocol-items-for-clinical-trials/).

Administrative information

**Table:** 

Title {1}	Reach Out behavioral intervention for hypertension initiated in the emergency department connecting multiple health systems: study protocol for a randomized control trial
Trial registration {2a and 2b}.	ClinicalTrials.gov Identifier: NCT03422718
Protocol version [1]	Version 9.0; January 17, 2020
Funding {4}	Funded by National Institutes of Health, National Institutes of Minority Health and Disparities R01 MD011516
Author details {5a}	William J. Meurer^1,2,3,4,5^; Mackenzie Dinh^1^; Kelley M. Kidwell^6^; Adam Flood^1^; Emily Champoux^2^; Candace Whitfield^1^; Deborah Trimble^1^; Joan Cowdery^7^; Dominic Borgialli^1,8^; Sacha Montas^1^; Rebecca Cunningham^1^; Lorraine R. Buis^4,9^; Devin Brown^2,3^; Lesli Skolarus^2,3^ *1. Department of Emergency Medicine, University of Michigan* *2. Department of Neurology, University of Michigan* *3. Stroke Program, University of Michigan* *4. Institute for Healthcare Policy and Innovation, University of Michigan* *5. Michigan Institute for Integrative Research in Critical Care (MCIRCC), University of Michigan* *6. Department of Biostatistics, School of Public Health, University of Michigan* *7. School of Health Promotion and Human Performance, Eastern Michigan University* *8. Department of Emergency Medicine, Hurley Medical Center* *9. Department of Family Medicine, University of Michigan*
Name and contact information for the trial sponsor {5b}	National Institute on Minority Health and Health Disparities (NIMHD) National Institutes of Health 6707 Democracy Boulevard, Suite 800 Bethesda, MD 20892–5465 Telephone: 301–402-1366 Fax: 301–480-4049 Email: NIMHDinfo@NIMHD.NIH.gov
Role of sponsor {5c}	The study sponsor and funders provided peer review of the study design. The sponsor had/will have no role in the collection, management, analysis, and interpretation of data; writing of the report; or the decision to submit the report for publication. They will not have ultimate authority over any of these activities.

## Introduction

### Background and rationale {6a}

Hypertension is the most important modifiable risk factor for cardiovascular disease, the leading cause of mortality in the United States. African Americans have the highest prevalence of hypertension of any racial or ethnic group in the United States contributing to their increased burden of stroke compared with non-Hispanic whites. In addition, uncontrolled hypertension is more common among socioeconomically disadvantaged populations than their counterparts.

The Emergency Department (ED) represents a unique opportunity to identify and impact hypertension in difficult–to-reach populations. Currently, there are 136 million ED visits per year and nearly all have at least one blood pressure (BP) measured and recorded. African Americans and socioeconomically disadvantaged patients are disproportionally represented within the ED patient population, and these proportions are increasing. In the age of electronic health records (EHR) and mobile health, the ED has the potential for integration as a partner in chronic disease management. Automatic programming through the EHR may be leveraged to identify hypertensive patients and dispense a mobile health behavioral intervention.

Primary care visits following an ED visit have been shown to decrease readmission rates, as well as improve health outcomes [1]. Particularly within socioeconomically disadvantaged populations, follow-up care after an ED visit is an area of interest for improvement; therefore, facilitation of follow-up at primary care clinics is a key feature of this intervention. Through utilizing the ED with its large patient volume of difficult-to-reach, uncontrolled, hypertensive patients combined with the continuity of care and chronic disease management expertise provided by the primary care clinics, we aim to improve community-wide utilization of health services.

### Existing and preliminary data

The scientific premise of Reach Out is to test an innovative, mobile health, behavioral, health system intervention to reduce BP in hard-to-reach, high-risk populations. An American Heart Association scientific statement on the use of mobile health for cardiovascular disease prevention found only 13 trials, of which only three, all performed outside of the US, used primarily text messaging to promote BP control [2]. Two of the three trials showed a reduction in BP but the results were limited by low participant retention and absence of intention-to-treat analysis [3, 4]. The American Heart Association’s scientific statement also identified several limitations in completed trials such as short durations of follow-up, especially less than 6 months, a lack of information on the optimal intervention components and their delivery, as well as an absence of rigorous clinical trial design [2]. Furthermore, many of the trials had only small reductions in BP, suggesting that incorporating text messaging into behavior interventions may be necessary for significant impact in underserved populations. Reach Out integrates behavioral approaches, such as self-monitoring, with novel strategies such as primary care provider (PCP) scheduling and transportation assistance to address these limitations [5–9].

Prior work by our team used a community-based participatory research (CBPR) approach, in which several mobile health components were developed including prompted BP self-monitoring, healthy behavior text messages, and a text messaging roadmap. Healthy behavior text messages and weekly prompted BP self-monitoring were then tested in three African American churches for 6 months to gauge suitability within the target population [10]. All text messages were delivered automatically through a messaging platform developed by Mosio. A total of 48 participants received the intervention with a mean age of 58 (standard deviation [SD] = 9.8) years, all of whom were African American. Notably, 15% of the participants did not have a PCP. Participants responded to BP prompts in 587 (47%) of the available 1248 person-weeks. All (100%) of participants reported satisfaction with the Reach Out intervention content. Overwhelmingly, participants did not want the mobile health intervention supplemented with phone calls, workshops, cooking demonstrations or internet modules.

We conducted a pilot feasibility trial of the healthy behavior text messaging and weekly prompted BP self-monitoring in the University of Michigan ED [11]. In this study, we created real-time automated alerts using the EHR to identify potentially eligible participants and procedures for recruitment of ED patients. Real-time automated EHR alerts were set to identify patients with systolic BP (SBP) ≥160 mmHG or diastolic BP (DBP) ≥100 mmHG. During the 7-month enrollment period, over 9300 patients with elevated BP were identified through the alerts. Because this was a pilot study that relied on volunteer undergraduate student research assistants, a convenience sample of 163 patients were approached and 104 patients were enrolled (64%), confirming the feasibility of automated real-time ED screening followed by enrollment of eligible patients. Of the 104 enrolled participants, 55 had at least one elevated BP during the screening period and were activated into the longitudinal portion of the study. Development and pilot testing of the intervention components evidenced feasibility; therefore, optimization is the next stage to determine which components and ‘dose’ of the components of the behavioral intervention are most effective.

### Overview of reach out

**Fig. 1:**
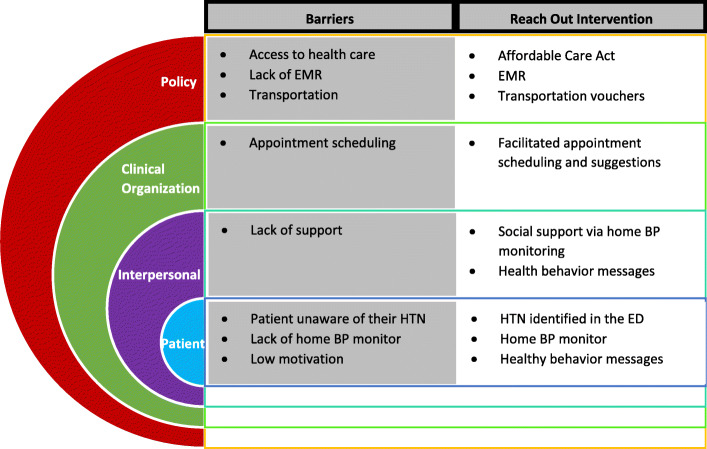
Reach Out and the social ecological model

Hypertension experts have called for the evaluation of multilevel interventions addressing barriers to hypertension care [7]. In line with this request and informed in part by the Social Ecological Model, Reach Out is designed to intervene at the individual patient, the health system, and the community levels (Fig. 1) [12] . At the individual level, Reach Out will advance the identification of hypertension through ED screening, providing BP self-monitors, and encouraging healthy behaviors, which are considered barriers to hypertension management in urban, under-resourced populations [7, 13–17]. At the health system level, Reach Out will work to reduce barriers by scheduling PCP appointments and will also assist with reducing the barrier of medication cost. For participants who establish care with the Federally Qualified Health Center (FQHC), medications are typically provided on a sliding fee scale, but additional guidance may be useful for some participants; therefore, healthy behavior text messages will be used to provide information about pharmacies with generic drug programs in the area. Additionally, Reach Out will work to reduce community barriers by providing free transportation to and from outpatient provider appointments.

### Objectives {7}

The Reach Out trial will determine the following:

Aim 1: Which behavioral intervention components or ‘dose’ of the components contributes to a reduction in systolic BP at 1 year.

Aim 2: The effect of PCP appointment scheduling and transportation on primary care follow-up of hypertensive patients initiated from an urban, safety net ED.

### Trial design {8}

Reach Out is a randomized, controlled, 2 × 2 × 2 factorial design clinical trial allocating subjects to one of eight available combinations of mobile health components (Table 1). An initial 3-week eligibility phase is used to assess for persistent hypertension and responsiveness to text messages. Participants are randomized into the main study if they have persistent hypertension (any self-reported SBP ≥140 mmHG or DBP ≥90 mmHG) and have submitted at least one BP by text during the screening period. If participants do not meet both criteria, they receive no further communication from the study team following the eligibility phase. A baseline assessment occurs at enrollment with outcome assessments at 6 and 12 months. The Reach Out program is designed to identify which mobile health components or ‘dose’ of components (healthy behavior text messaging, prompted BP self-monitoring and facilitated PCP appointments with transportation) contribute to a reduction in BP among hypertensive participants recruited from an urban, safety net ED (Table 2).

**Table 1:** Allocation of intervention components by arm

Arm	Healthy behavior text	Prompted BP self-monitoring frequency	Facilitated Primary Care appointment scheduling and transportation
1	No	Low	No
2	Yes	Low	No
3	No	High	No
4	Yes	High	No
5	No	Low	Yes
6	Yes	Low	Yes
7	No	High	Yes
8	Yes	High	Yes

*BP* blood pressure

**Table 2:** Intervention components and relationship to BP

Texts	Comparison levels	Tailoring variables	Mechanism for BP reduction
Healthy behavior text	Daily vs. none	-No	-Decrease salt intake -Increase physical activity -Increase fruit and vegetable intake
		-Medical provider -BP medication -Self-efficacy	-Discuss with provider -Increase medication adherence
Prompted BP self-monitoring frequency	Daily vs. weekly	-BP change (most recent self-reported BP) -BP control	-Participant activation -Participant autonomy -Participant competence
Facilitated Primary Care appointment scheduling and transportation	Yes vs. none	-Medical provider -BP control	-Improve access to medical care -Opportunities for medication optimization

*BP* blood pressure

## METHODS: Participants, interventions and outcomes

### Study setting {9}

This trial takes place in Flint, Michigan, an urban, underserved, predominately African American community. Flint has a population of 102,434, of which 57% are African Americans and 37% live below the national poverty level [18]. On US national surveys, approximately 40% of Flint residents have self-reported a diagnosis of hypertension [19]. The Hurley Medical Center, a 540-bed hospital, is the only Level 1 Trauma Center safety net teaching hospital located in Flint, MI (Genesee County). The Hurley ED provides care for approximately 110,000 patient visits per year and recently has more than doubled its ED space, making it ideally situated to facilitate the Reach Out study. To initiate PCP appointments for patients who do not already have one, the study is partnering with the Hamilton Community Health Network, which is an FQHC outpatient care network in Flint, caring for over 30,000 patients, about 65% of whom are African American.

### Eligibility criteria {10}

#### Inclusion criteria

- Age of 18 or greater
- At least one BP with SBP ≥ 160 mmHG or DBP ≥ 100 mmHG (Criterion 1)
- If the patient has repeated measurements after achieving Criterion 1, at least one of the repeat BPs has a SBP ≥140 mmHG or DBP ≥90 mmHG
- Must have a cell phone with text-messaging capability and willingness to send and receive texts
- Likely to be discharged from the ED (not already admitted to hospital and medical provider from clinical team anticipates home disposition)

#### Exclusion criteria

- Unable to read English (< 1% anticipated at study site)
- Prisoner
- Pregnancy
- Pre-existing condition making 1-year follow-up unlikely
- Terminal illness with death expected within 90 days
- Current use of three or more antihypertensive agents
- Patients with other serious medical conditions that prevent self-monitoring of BP
- Critical illness with placement in resuscitation bay
- Dementia/cognitive impairment

### Who will take informed consent? {26a}

The study team members will provide information about the study and obtain consent. The clinical team caring for the patient in the ED is not involved in the consent process.

### Additional consent provisions for collection and use of participant data and biological specimens {26b}

Reach Out does not collect biological specimens. Should ancillary studies be Institutional Review Board (IRB)-approved that require the collection of additional data or any specimens, a separate consent process will be used, or a check box added to the present consent form with additional information.

### Interventions

#### Explanation for the choice of comparators {6b}

Reach Out tests three intervention components, each with two possible intensity levels: (1) healthy behavior text messaging (daily vs. none); (2) prompted BP self-monitoring (daily vs. weekly); and (3) facilitated primary care provider scheduling and transportation (yes vs. no). Following a 3-week screening period, participants are randomized into one of the eight arms (Table 2) for the duration of the 12-month intervention. There is no “untreated” control group because the efficacy of prompted blood pressure self-monitoring and feedback has been demonstrated in preliminary studies. Therefore, as the lowest intensity group, Arm 1 serves as the control. This arm is comprised of weekly prompted self-monitoring and reporting of BP with no healthy behavior messages or facilitated PCP scheduling and transportation.

#### Intervention description {11a}

##### Intervention 1: Healthy Behavior Text Messaging

Participants are randomized into either daily texts or no texts arm. Our team, partnering with the community, created the content for these text messages based on social cognitive theory with an emphasis on self-efficacy, outcome expectations, knowledge, social support, and reinforcement. The messages provide strategies for the following important lifestyle interventions to reduce BP: salt reduction, increased fruit and vegetable intake, and increased physical activity [20–23]. Text messages are both tailored and targeted to increase the personal relevance and therefore the efficacy of the behavioral interventions. Targeting refers to the creation of materials focused on shared characteristics of a group of people such as messaging relevant to participants taking medications. Tailoring, on the other hand, refers to materials focused on characteristics of individuals such as feedback on their own previous BP inputs. Participants receive a text message each week tailored to a recent self-reported BP compared against normal BP thresholds, 130/80 mmHG, targeted to provider, medication, and medication adherence. Variables that direct the targeting and tailoring messages are reassessed at several points throughout the duration of the study.

##### Intervention 2: Prompted BP Self-Monitoring

**Fig. 2:**
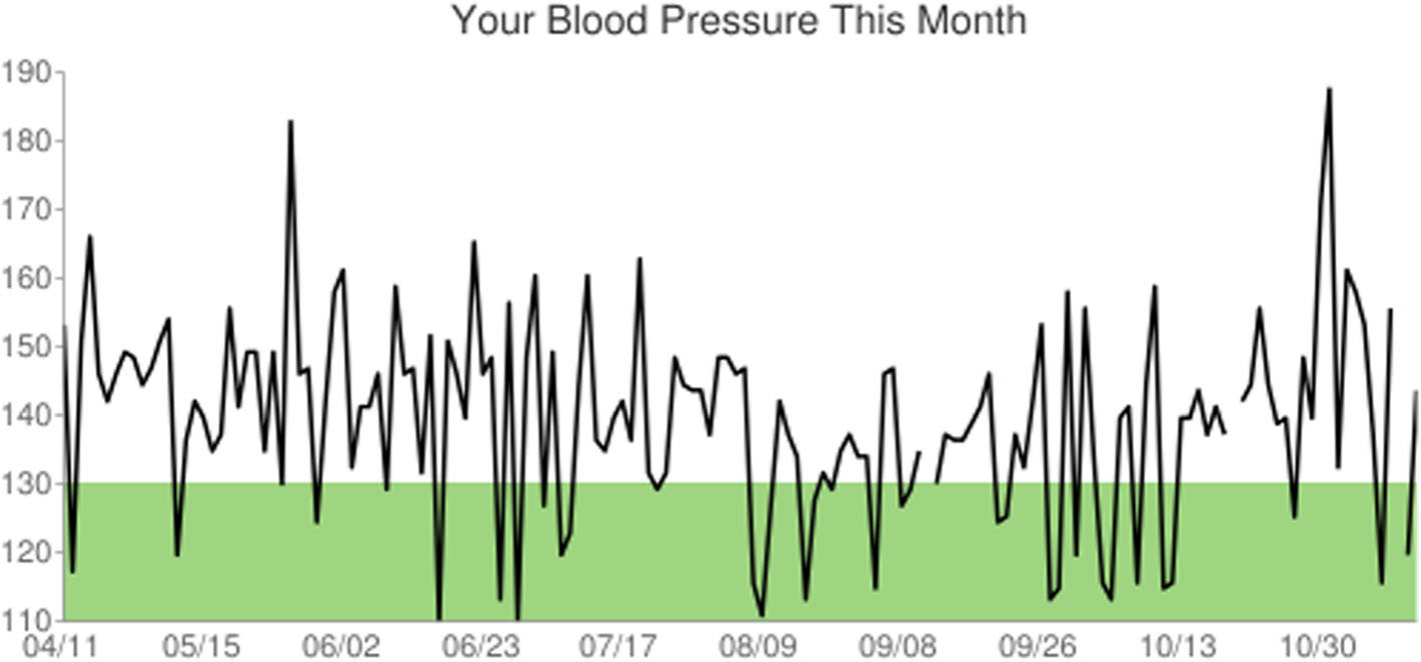
Monthly feedback graph example

Again, participants are randomized initially into either a high-intensity (daily texts) or low-intensity (weekly texts) arm. A BP self-monitoring device is distributed at enrollment in the ED along with training for cuff use and text-message response formatting. BP inputs are then requested at daily or weekly intervals depending on randomization. The messaging system additionally benefits participants by providing a longitudinal record of BP readings that can be submitted at PCP appointments for use in the chronic management of hypertension. As one form of feedback, participants receive a tailored message each week based on a recent self-reported blood pressure compared against normal BP thresholds, 130/80 mmHG. Additionally, participants may prompt the text messaging system to provide a graph or list of their self-reported BPs since their randomization. The second form of automatic feedback is a monthly graph of self-reported SBPs (Fig. 2). Our platform allows the participants to provide preferences regarding the time of day for the BP reminders, as well as the ability to adjust these as needed. The text-messaging system sends three BP prompts per week if the participant does not initially respond with a BP.

Participants who have a BP > 180/120 mmHG are sent an automated message telling them to call their doctor right away as they likely need adjustments to medication or if symptomatic with chest pain, weakness, difficulty talking, or confusion, to call 911. If a message is sent to the system that is not in the format of a BP reading, an automated error message is sent back to the participant for them to resubmit their BP in the previously taught format. Participants have the option to modulate their own reminder frequency by texting prompts to receive daily BP reminders, weekly reminders, or revert to the original frequency at randomization.

##### Intervention 3: PCP appointment scheduling and transportation

Recognizing the importance of PCP visits, Reach Out is working to improve outpatient preventive care. Study participants are randomized to receive either facilitated scheduling and transportation or passive reminders to schedule their own PCP appointments. For participants without a PCP, outpatient care is established at a Hamilton Community Health Center within approximately 6 weeks of discharge from the ED. For participants randomized to the facilitated group, the research team identifies up to three available PCP appointments and text those options to the participant for their selection. If a participant does not select an appointment time out of the three provided options, a member of the research staff may call to assess barriers with the participant.

Preceding the appointment, text messages are sent to the participant to remind him/her of the appointment. Participants are offered transportation to their appointments, which is most readily available in their neighborhood (taxi, public transportation, or ride share services). If necessary, a study team member may contact the participant via phone call or text message during this process to clarify or complete appointment scheduling. These appointment and transportation procedures occur monthly (on average) until BP control is achieved. Following BP control, which is defined as SBP < 130 mmHG, the frequency of recommended provider visits decreases to approximately every 3 months.

Participants who are randomized not to receive facilitated PCP appointment scheduling and transportation receive monthly text messages encouraging them to contact their PCP to schedule an appointment. Transportation is not provided in this study arm, with the exception of outcome assessments for the study at 6 and 12 months.

#### Criteria for discontinuing or modifying allocated interventions {11b}

Participants have the option to de-escalate, pause, or completely stop the text messages and withdraw from the messaging portion of the study. We collect outcome assessments even if the participant stops receiving text messages unless there is a request to completely withdraw from the study.

We are not enrolling any of the following vulnerable populations: pregnant women, children, or prisoners. Because hypertension is a different disease in pregnancy, potential participant’s pregnancy status is verified by either a negative pregnancy test performed in the ED or specific confirmation by research staff during recruitment screening. There is no specific risk for a pregnant woman; however, the results would not be comparable to other study participants given the different pathophysiology of hypertension in pregnancy.

Once enrolled, participants of childbearing age are instructed to notify the research team if they become pregnant. Additionally, pregnancy status is confirmed at the 6-month outcome assessment. If the research team learns that a participant is pregnant at the research visit or is notified at another time during the study, the intervention elements are discontinued and, if desired, the team will work with the participants’ PCP to help establish prenatal care. In general, by the intention-to-treat principle, pregnant women will be kept in their cohort to monitor and collect outcomes, but the active intervention components will not be used.

Prisoners would not generally have access to the ability to text, keep a BP monitoring cuff, or utilize appointments with outside PCPs and thus would not be eligible to participate in the study interventions. Enrolled patients may become prisoners while in our study. The study team does not actively screen for this; however, if we learn this during the course of participation, we will place study procedures on hold while the participant is a prisoner. Participants may resume activity in their assigned intervention after the period of incarceration, provided the 1-year follow up period has not been expended. We will not extend the follow-up window in these cases.

#### Strategies to improve adherence to interventions {11c}

Participants are given a blood pressure cuff at enrollment (about USD 20 value) plus USD 20 at the completion of the baseline data collection. In addition, during recruitment in the ED, we provide an educational comic book about hypertension (Additional file 1), instructions for text messaging (Additional file 2) and instructions for taking the BP (Additional file 3) to ensure that participants have appropriate reference materials for participation in the trial.

Participants are given USD 25 for completing the 6-month outcome assessment and USD 30 for completing the 12-month outcome assessment. Participants randomized to receive facilitated PCP appointment scheduling are also provided transportation for their appointments (about USD 15–30 per ride). All participants are offered transportation for the study outcome assessment appointments. We track all incentives allocated to participants through their respective intervention arms. At enrollment, participants are given a variety of items designed to help promote enthusiasm for the project including nominal gifts such as a pen, a bag, and other assorted materials with the Reach Out logo.

#### Relevant concomitant care permitted or prohibited during the trial {11d}

The medical care of the participants will be determined by their clinical providers. There are no prohibited concomitant treatments.

### Provisions for post-trial care {30}

The participants keep the blood pressure cuff; however, the participants do not receive the study-related interventions after the completion of participation.

#### Outcomes {12}

##### Endpoints – primary and secondary

The primary endpoint is any change in systolic BP from baseline screening to 12 months (if more than one BP reading is available for the screening baseline then the median will be used). There are two secondary endpoints: (1) number of days between ED visit and self-reported arrival at first PCP visit and (2) self-reported attendance at two or more PCP visits within 12 months of randomization.

##### Exploratory endpoints

BP

- Proportion of participants achieving BP control
  - Eighth Joint National Committee (JNC-8) targets for BP (< 140/90 mmHg)
  - 2017 American Heart Association (AHA) guidelines targets for BP (< 130/80 mmHg)
- Change in DBP and mean arterial pressure
- Change in 6-month SBP

Process

- Number of interactions (such as calls/texts) required to schedule participants’ initial PCP visit
- Number of interactions to schedule all additional PCP visits
- Proportion of responses to BP request messages by the study system
- Proportion of participants enrolled but not randomized

PCP

- Establishment of a PCP among those without
- Number and proportion of PCP visits attended
- Proportion of transportation vouchers used

Utilization

- Frequency and number of Hurley ED and other healthcare visits
- Self-reported follow-up within the Flint health system – Hurley, Hamilton, or community clinics
- Follow-up outside of the Flint health system – urgent care, outside clinics, outside EDs

Medications

- Self-reported changes in antihypertensive regimen
- Medication adherence (Hill-Bone Scale, and modified Hill-Bone instrument asking, “How often do you forget to take your BP medicine?”)

Other

- Social Cognitive Theory (SCT) measures
  - Modified one-question self-efficacy measure, “I am confident that I can take my blood pressure and text it to the Reach Out Team”)
  - Questions querying self-efficacy, motivation, social support, and expertise.
    - I am confident that I can control my blood pressure
    - It is worthwhile for me to control my blood pressure
    - My friends and family care if I control my blood pressure
    - I know the right steps to take to control my blood pressure
- General intervention feedback
  - Acceptability of Intervention Measure (AIM)
  - Feasibility of Intervention Measure (FIM)

##### Validity of the primary endpoint

In meta-analysis estimates for a given BP, SBP predicted over 90% of the risk of ischemic heart disease and stroke [24]. In other studies, a 2 mmHg decrease in SBP has been shown to have the potential to reduce stroke mortality by 10% and ischemic heart disease mortality by 7%. This provides a clear justification that the continuous outcome of change in systolic BP is clinically relevant.

#### Participant timeline {13}

**Fig. 3:**
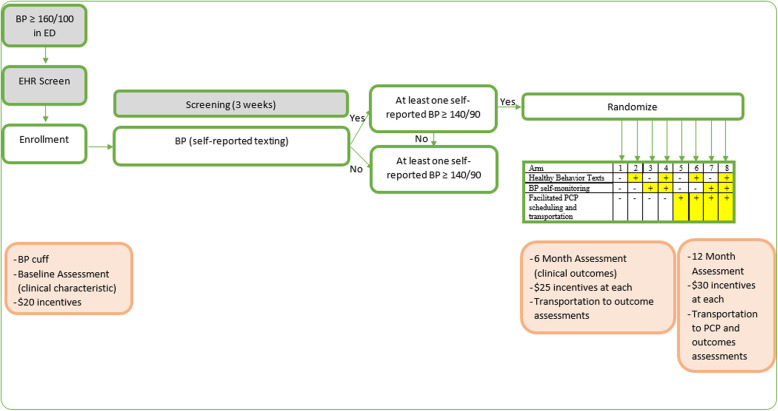
Overview of reach out design

Due to the prevalence of isolated hypertension in the ED, participants undergo a 3-week eligibility phase to determine if they have persistently elevated BP outside of the events that brought them to the ED (Fig. 3). All participants enrolled in the study are given an automated BP cuff, AHA or American College of Cardiology information about high BP, a Reach Out comic book, and will receive a weekly text message asking their BP. If a participant has not responded by the end of week two, a study team member may call them up to two times to assure it is not a technical issue. This additional level of contact is based on feedback from our previous studies, where participants’ most cited reason for unresponsiveness was unaddressed technical complications.

**Fig. 4:**
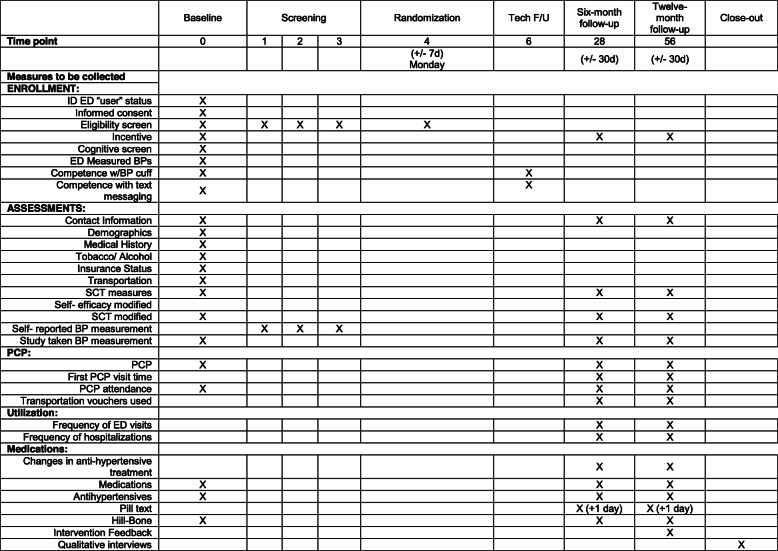
Overview of study timeline. (Meds/ PCP survey and modified Hill Bones are collected every 4 weeks)

Participants are randomized into the main trial if they have at least one elevated blood pressure (SBP ≥140 or DBP ≥90) and have responded to text messaging at least once during the 3-week screening period. If participants do not meet both criteria, they receive no further communication from the study team following the eligibility phase aside from a final text message thanking them for their participation. For participants randomized to a treatment group, study participation will continue for 12 months. (Study procedures and visits are summarized in Fig. 4).

#### Sample size {14}

We plan to enroll approximately 1440 patients into the eligibility phase. From this group, we estimate that 720 participants will report qualifying BPs and be randomized to one of the eight intervention arms. We anticipate 240 participants will complete the full study including the 12-month in-person follow-up visits. Collins and others have reported on methods for sample size estimations for factorial design trials included in the multiphase optimization strategy framework [25, 26]. It is important to note that the primary hypotheses driven by these designs were derived from the overall estimate of the effects of each of the individual intervention components and their combinations rather than the pairwise comparisons of each of the interventions. In order to represent a clinically significant result, the minimum required BP change for each component will be set at 1 mmHg with a standard deviation of 2.8 mmHg, corresponding to a standardized effect size of 0.35*.*

We require 196 randomized participants in total in order to detect, based on the Collins method of a three-factor experiment analyzed using a second order model (i.e. main effects and two-way interactions), the minimum standardized effect of 0.35 at 80% power. Assuming 720 randomized participants and allowing more than 50% loss to follow-up after the eligibility phase, we anticipate more than 240 participants will complete the study, which exceeds the required number for 80% power with an alpha level of 0.10 (inflated to reduce the likelihood of dismissing active components with modest BP reducing effect). We used the Factorial Power Plan SAS macro (SAS Institute Inc., Cary, NC, USA) to calculate power.

For analysis of the secondary objective, using an alpha of 0.05 and a baseline assumption of 40% attendance at two or more PCP visits within the follow-up year, a total sample size of 240 is needed, which will be evenly distributed between the active appointment scheduling and passive reminders groups. Under these assumptions, we have 87% power to detect an increase to 60% (from 40%) attending two or more visits. The time-to-event analysis evaluating the time to first PCP visit after randomization (considering an alpha of 0.05, power of 99%, and 240 participants enrolled over 720 days, followed for 30 days) can detect a change from 20% of participants in the non-facilitated group to 20.4% of participants in the facilitated group. This high power allows ample buffer for covariates and loss to follow-up. Sample size calculations for secondary outcomes were performed with STPLAN (version 4.5 available from M.D. Anderson Department of Biostatistics at their software download kiosk – https://biostatistics.mdanderson.org/SoftwareDownload/SoftwareDownload/Index/). The preliminary statistical analysis plan is available as a supplementary material (Additional file 4).

#### Recruitment {15}

We implement an automated screening algorithm used by the EHRs of the ED, to identify patients with BPs meeting the enrolment threshold of SBP ≥160 mmHG or DBP ≥100 mmHG. This screening threshold may be adjusted if the proportion of patients with persistent hypertension in the screening phase is less than 40% or more than 80% of enrolled patients. This system automatically alerts the study team member(s) that a patient meets the BP eligibility criteria and should be further screened for potential enrolment.

Patient BPs are obtained during routine patient triage at all ED visits. If the alert system is not functional at any point, it may be supplemented by manual chart review of the daily ED patient log. The automated identification system may commence up to 3 months prior to the opening of study enrolment to address errors and optimize the EHR screening algorithm within Hurley. We were granted a waiver of consent to maintain a screening log of patients that trigger the alert system, and through chart review, collect the following demographics: age, gender, race/ethnicity, reason for ineligibility or refusal, and ED BP.

All adults meeting the inclusion/exclusion criteria are approached for consent if a study team member is available. Following initial screening through EHR for BPs meeting inclusion criteria in the ED, an in-person screening of potential participants is conducted based on remaining inclusion/exclusion criteria. Cognitive ability is assessed through the question, “Are you able to stay home alone for 24 hours?” After consent, baseline characteristics are collected, including the data needed for targeting and tailoring future text messages. Participants receive a validated, automated, oscillometric BP monitor (SureLife Classic Wrist Blood Pressure Monitor or similar [MHC Medical Products, LLC, Fairfield, OH, USA]) and USD 20 compensation for their time. The research staff teaches the participants how to use the BP cuff including the timing of BP self-monitoring, body position, and resting prior to testing, as well as, how to text the BP readings.

### Assignment of interventions: allocation

#### Sequence generation {16a}

Participants who provide informed consent and continue to be eligible during the 3-week screening phase assessing for persistent hypertension and responsiveness to text messages will then be randomized. Participants will be randomized via 2 × 2 × 2 block randomization into one of the eight experimental arms of the main trial. Each arm contains one of the two intensity levels of the three interventions (Table 1). The sequences are generated using a random number generator and uploaded into a system called TATUM by the unblinded statistician.

Because the intervention components may have important associations with participants’ baseline characteristics, randomization into study arms is stratified by age, sex, and antihypertensive use. Specifically, in order to ensure scientific rigor and reduce imbalance within our treatment arms the stratification variables are age (< or ≥ 65), sex (male or female), antihypertensive use within the last 6 months (yes or no).

#### Concealment mechanism {16b}

Randomization into the stratified study arms occurs in the central office. Computer software is used for this process, preventing the study staff from knowing the next sequence. Given the distribution across eight different combinations of intervention components, it is unlikely the research staff could predict the next number. In addition, since participants have a 3-week eligibility phase prior to randomization, it is not possible for the enrolling staff to know what treatment group will be assigned to any specific individual 3 weeks later.

#### Implementation {16c}

The allocation sequence was generated by the study statistician and tested in the online randomization system. The principal investigators and enrolling study staff are unaware of the sequence.

### Assignment of interventions: Blinding

#### Who will be blinded {17a}

There is no blinding to intervention groups in this study. Although at the time of recruitment, study staff are unaware of the future treatment group assignment following randomization, study staff are aware of the treatment group assignment because they are facilitating transportation and troubleshooting text messages for participants. Patients are also aware of their treatment group assignment, as they will be told if they are to receive transportation assistance or not and may note the frequency of BP checks or presence or absence of healthy behavior texts.

While the trial is ongoing, the principal investigators and study staff will be blinded to the primary endpoint analysis of each group in order to decrease bias toward a particular group. This requirement will be reviewed routinely by the trial statistician and, the independent medical monitor, if requested.

#### Procedure for unblinding if needed {17b}

An unblinding procedure is not applicable for this study as participants will be aware of their treatment group assignment after randomization. If the independent medical monitor determines the principal investigators should be unblinded to differences in final outcomes across groups, he or she will first notify the study statistician and the National Institute on Minority Health and Disparities (NIMHD).

### Data collection and management

#### Plans for assessment and collection of outcomes {18a}

The primary endpoint is the change in SBP from baseline to 12 months; therefore, BP will be measured at every in-person contact. A baseline assessment will occur at enrollment and outcome assessments at 6 and 12 months. At baseline, the BP recorded will be the BPs as documented by the medical staff during the ED visit. At 6- and 12-month outcome visits, BP will be measured by the research team using a validated BP cuff, in accordance with national standards for measurement. Outcome visits may occur at a provider office to coincide with a scheduled provider appointment, in the ED research space, or in a mutually convenient location (e.g., home, library, or restaurant).

#### Plans to promote participant retention and complete follow-up {18b}

The challenges of recruiting racial minorities and people with socioeconomic disadvantage for clinical research studies are well documented. Similar challenges are notable for retention in research. Reach Out participant recruitment is facilitated by several factors: (1) the study is minimal risk; (2) culturally and community sensitive recruitment materials were created using CBPR principles); (3) enrollment via an urban, safety net ED; and (4) monetary incentives.

At the 6- and 12-month assessments, text messages and/or phone calls are sent to all participants to facilitate the outcome assessment scheduling with a reminder text two- and 1 day before the appointment. A transportation credit will be offered to participants via a driving service available in their targeted neighborhood (taxi, public transportation, or ride share) in order to maximize attendance at in-person outcome visits.

#### Data management {19}

The main study database platform is Research Electronic Data Capture (REDCap), all data is entered electronically. A subset of study participant data outcomes will undergo additional review to assess data quality. A second assessor will review a random subset. Quality checking will assess original sources for inconsistency, checking other sources to determine the correction, and modify the original form. Written documentation of changes will be available via electronic logs or audit trails. Based on these results, abstractors may attend a refresher training to improve accuracy. Range checks and content validation will be utilized in the database to ensure high-quality data entry. When applicable, source documents will be uploaded for remote verification.

#### Confidentiality {27}

The main risk to patients in this study is a potential breach of confidentiality of medical records and survey responses that could result in psychological distress. The likelihood of this risk is estimated to be rare and the seriousness to the participant is estimated to be low. To prevent this occurrence, data obtained from participants is considered confidential and stored in locked facilities and password-protected computers. Trained abstractors will enter pertinent information directly into the REDCap database, a password-protected web-based application designed to securely store identifiable information. The text messages are transmitted within a Secure Sockets Lay encrypted web-based application with secure logins to access. A data use agreement has also been established with the text messaging vendor. Additionally, the text message company underwent a security review by the University of Michigan for safety standards and protections. We have obtained a Certificate of Confidentiality from the National Institutes of Health permitting legal refusal to disclose information that may identify a participant.

#### Plans for collection, laboratory evaluation and storage of biological specimens for genetic or molecular analysis in this trial/future use {33}

Reach Out has no plans for collection or storage of biological specimens.

### Statistical methods

#### Statistical methods for primary and secondary outcomes {20a}

The formal statistical analysis plan was codified prior to enrollment and is available in the supplementary material (Additional file 4). The primary analysis (Aim 1) will fit a linear regression model with the outcome of SBP change (baseline minus 12 months) and main effect encoded as binary products of healthy behavior texts (yes vs. no), prompted BP self-monitoring frequency (high vs. low), and PCP visit scheduling and transportation (active vs. passive). Initial analyses will focus on the main effects. Additional analyses will include all the two-way interactions of the three intervention components, focusing on interactions where at least one of the factors in the interaction demonstrates a sufficiently large main effect. As the goal of this exploratory trial is to find interventions or combinations that improve BP control, we will use an alpha level of 0.10 for all main effects and 0.20 for interactions. We plan to include elements meeting this threshold for statistical significance in a subsequent multicenter trial. If significant interactions exist (e.g., the combination of facilitated transportation and high frequency home BP monitoring), the future study will assess the combination of elements that has the highest expected reduction in SBP – assuming we achieve significance at the *p* = 0.1 (main effects) or 0.2 (interaction) level for at least one component. In the event of an overall “null” trial, we would re-examine the expected change in SBP for Arm 1, represented by the intercept. If this was significantly greater than 0 at the *p* = 0.1 level, we will propose a subsequent multicenter trial using only weekly prompted BP self-monitoring.

The main secondary analyses (Aim 2) will use logistic regression to model attendance at two or more PCP visits within 1 year, and time-to-event (Cox proportional hazards) to model time to first PCP visit. For the endpoints of interest, time to first PCP visit or a binary variable indicating attendance at two or more PCP visits within 1 year of randomization, we will fit an adjusted regression model. The main predictor of interest for these models is assignment to the active PCP scheduling and transportation arm (50% of all participants randomized in the trial), while adjusting for the change associated with assignment into other groups, such as healthy behavior text messages and prompted BP self-monitoring, as in Aim 1.

We will conduct additional exploratory analyses using the randomization stratification factors of age, sex, and anti-hypertensive use as covariates. As uncertainty exists about the distribution of enrolled participants within these strata (e.g., young men taking antihypertensive medication), we will likely not be powered for these exploratory aims. Interaction terms (e.g., age or sex with behavioral interventions, etc.) will be utilized to determine if specific groups respond better than others to specific intervention elements. Baseline BP may also play an important role, which will be examined by dividing the cohort into terciles. We will repeat the above primary and exploratory modeling within each of the three initial BP strata to determine if there is heterogeneity of effect, such as whether patients with the highest baseline BPs get the most benefit from the intervention components. We will similarly assess the impact of the intervention components on the exploratory and process outcomes using linear, Poisson, logistic, or ordinal regression depending on the distributions. In addition, we will conduct longitudinal analyses that include all BP measurements over time (including those by text and at the in-person visits) to assess the temporal profiles of response in the intervention components. The detailed system application and product (SAP) in data processing outlines procedures for handling missing outcome data.

#### Interim analyses {21b}

The trial does not employ formal efficacy or futility stopping rules. Interim data will be reported to the independent medical monitor according to the charter approximately every 6 months during active recruitment.

#### Methods for additional analyses (e.g., subgroup analyses) {20b}

Subgroup analyses aim to evaluate the heterogeneity of treatment effects according to baseline characteristics of age, sex, previous antihypertensive medication, race, and baseline BP level. These are defined in detail in the statistical analysis plan.

#### Methods in analysis to handle protocol non-adherence and any statistical methods to handle missing data {20c}

Approaches to missing data are outlined in the SAP. Briefly, the interim self-reported BPs will be used, if necessary, to impute final SBP using established methods. Regarding protocol non-adherence, we will conduct weekly sensitivity analyses focused on the group assignment of each participant to monitor their progress through the assigned arm or transfer request to an arm with different texting frequency.

#### Plans to give access to the full protocol, participant level-data and statistical code {31c}

The full protocol at the time of initial submission of this manuscript is available on request. The final protocol and SAP will be uploaded to clinicaltrials.gov at the completion of the trial and will be provided as Appendix to the main result manuscript. After de-identification, we plan to share the individual level data and statistical code on the University of Michigan institutional repository, Deep Blue Data. We plan to have this ready concurrent with submission of the primary paper. In addition, we plan to post the primary paper to a compliant pre-print server prior to submitting to a peer-reviewed journal so the results can be immediately available to the public; if needed by the copyright requirements of the target journal, we may embargo this posting until acceptance of the primary paper.

### Oversight and monitoring

#### Composition of the coordinating center and trial steering committee {5d}

The principal investigators, project manager, and enrolling research staff comprise the operations committee at the coordinating center. The principal investigators, the additional co-investigators, consultants, and the biostatistician comprise the trial steering committee.

#### Composition of the data monitoring committee, its role and reporting structure {21a}

A data monitoring committee is not needed for this trial given the low risk use of existing technologies to help control BP. In collaboration with NIMHD we set up independent oversight through a medical monitor. An independent medical monitor (IMM) has the responsibility to monitor data and oversee participant safety: they will be approved by the NIMHD for oversight of the trial. The Reach Out IMM will be an expert in the care of hypertension and may either be an emergency medicine physician or a primary care physician. The frequency of IMM meetings will be determined by the IMM, funder, and principal investigator (PI). The IMM will review data on outcomes, accrual, and adverse events.

#### Adverse event reporting and harms {22}

An adverse event (AE) is any unfavorable and unintended sign, symptom, or disease temporally associated with the use of a medical treatment or procedure regardless of whether it is considered related to the medical treatment or procedure (attribution of unrelated, unlikely, possible, probable, or definite). Each AE is a unique representation of a specific event used for medical documentation and scientific analysis. A serious adverse event (SAE) is an AE that is fatal or life threatening, is permanently or substantially disabling, requires or prolongs hospitalization, results in a congenital anomaly, requires intervention to prevent permanent impairment or damage, or any event that the treating clinician or internal medical monitor judges to be a significant hazard, contraindication, side effect, or precaution. Reporting serious adverse events (SAEs) are based on the guidelines of the International Conference on Harmonization (ICH). We will only track adverse events definitely, probably, or possibly related to the intervention.

All SAEs occurring during study participation will be documented on the AE case report form. Adverse events will be documented using the NCI Common Terminology Criteria for Adverse Events Version 3.0 (CTCAE). The CTCAE provides descriptive terminology that will be used for recording and reporting AEs that occur. At the time that an AE is reported, the PI is responsible for designating the likelihood that the AE is caused by the study intervention. This determination requires clinical judgment but for purposes of this study, an algorithm is used to help the investigator report in a manner that is consistent across the trial and as objective as possible.

All AEs and SAEs reported to the research team that occur during participation in the study are recorded on the AE case report form per IRB policy. The PI, study coordinator or designee is responsible for entering all AEs and SAEs into the database as soon as he/she becomes aware of the event and updating that information (e.g., date of resolution, action taken) in a timely manner. All non-serious AEs must be recorded on the electronic AE case report form within 5 days from the time it was discovered by the study personnel. All SAEs and non-serious AEs must be entered by the end of study for that participant.

#### Frequency and plans for auditing trial conduct {23}

The investigators and the project manager will conduct a monthly audit of screening, enrollment, and data entry. Overall conduct will be monitored by the IMM who will receive summary data on adverse events and outcomes.

#### Plans for communicating important protocol amendments to relevant parties (e.g., trial participants, ethical committees) {25}

Any important protocol modifications (e.g., changes to eligibility criteria, outcomes, analyses) will be reported promptly to relevant parties: investigators, IRBs, trial participants, trial registries, journals and regulators. Administrative protocol changes that do not impact relevant parties or registries will be reported annually.

#### Dissemination plans {31a}

We plan to share the study results through presentations at scientific meetings, manuscripts, and community meetings. In addition, we will send a results summary to current and completed participants who opted in to receive study information, updates, or final results of the study at the completion. The study was registered in ClinicalTrials.gov prior to the enrollment of the first participant and is publicly registered with identifier NCT03422718. Results information from the study will be submitted to clinicaltrials.gov no later than 1 year after the trial’s primary completion date. The informed consent documents include a specific statement outlining intent to post clinical trial information at ClinicalTrials.gov. Additionally, the University of Michigan has internal policies, supported by the Office of Regulatory Affairs, to ensure clinical trial registration and results reporting occur in compliance with policy requirements.

## Discussion

Traditionally, multicomponent interventions are determined a priori and then the entire intervention is tested in a randomized controlled trial (RCT). RCTs are limited when evaluating multicomponent behavioral interventions because data are limited or non-existent regarding the performance of individual intervention components. As a result, it is unknown whether each component’s effect size justifies the resources required to implement it [25, 27]. Traditionally, the cycle of intervention refinement includes intervention → RCT → post hoc analyses → revision of intervention → RCT. This process is slow, costly, susceptible to multiplicity due to the number of subgroups, and leads to little quantitative optimization [28]. Prior text messaging-based interventions have had mixed results, although meta-analyses generally favor positive effects. Neutral or negative trials may have been underpowered or potentially mis-specified their intervention regimen with either too few components (akin to an inadequate dose in drug discovery) or too many components (inducing intolerable annoyance akin to dose-limiting side effects of a medication).

An alternative to traditional two-arm trials comparing multicomponent interventions to a control is the Multiphase Optimization Strategy (MOST), a three-phase strategy adapted from an engineering framework [29, 30]. The first stage, preparation, includes development and pilot testing of the intervention components based on a theoretical model. Optimization is the second stage during which data are gathered, often using factorial design, to determine which components and ‘dose’ of the components of the behavioral intervention are most effective. Factorial designs allow for an estimate of the individual effects of the components, the component interactions between groups and whether they are an efficient use of time, money, or resources [26, 31]. The third stage, the evaluation phase, determines whether the optimized intervention is effective using an RCT [30]. It may be that there is no combination of intervention components likely to be effective after optimization testing, at which time the study would not move on to the evaluation phase but instead move back to the preparation phase. Reach Out has completed the preparation phase and is now undergoing optimization testing. The factorial design within the MOST framework is crucial because we can learn which components, if any, are most likely to succeed in future trials.

While not a traditionally defined vulnerable population, the people of the city of Flint have faced unique challenges recently with the water crisis. The University of Michigan Department of Emergency Medicine has a long-standing relationship with Hurley Medical Center and the city of Flint. Our faculty group has staffed the ED there for almost 20 years. We have an active research infrastructure on location working to reduce the burden of substance abuse and violence in this community. We anticipate that the community will be distrustful of government-funded research and we will therefore be extremely diligent in working with our community partners and employing research staff sensitive to diverse populations. This research study involves limited risks to the community and is truly designed to provide methods of improving access to hypertension treatment, so it is unlikely to provoke controversy.

### Potential challenges

We may observe few differences between higher and lower levels of intervention components: it is possible that there will be little or no effect with the increased intensity intervention components. In this scenario, given the established benefits of BP self-monitoring in the general ambulatory population, we would pursue a large, simple trial of prompted BP self-monitoring in a population of ED patients [5, 6, 8, 9, 32]. Regardless, our overall development approach will allow us to examine the relationship and overall impact of each of our predictors and interactions with clinical and demographic variables. Costs of purchasing BP cuffs present a potential barrier to widespread implementation; however, a recent cost-benefit analysis found that BP self-monitoring saved money long term and coverage is supported by major medical societies [33].

### Generalizability

Similarly, there are over 1000 FQHCs with locations in every state. Safety net hospitals and FQHCs are often co-located in underserved areas where post-ED outpatient follow-up is not typically prioritized. EPIC, the EHR used at Hurley Medical Center, is the largest provider of EHRs in the country, further supporting the generalizability of Reach Out. Finally, 80% of Americans have text messaging capability. Given the ubiquity of Reach Out components, it could be implemented nationwide.

### Impact

Reach Out has the potential to serve as a model for safety net health systems to optimize hypertension care among their patient populations. The pragmatic design of Reach Out including broad enrollment criteria, use of the EHR to identify potential participants, and use of technology for health intervention. These key features will allow for future implementation of the trial components in real-world health systems.

## Trial status

Recruitment started on March 25, 2019. As of January 16, 2020, we had enrolled 768 participants in the eligibility phase, of whom 398 have been randomized. The approximate date of recruitment completion is November 1, 2020. The current protocol version approved by the IRB is 9.0.

### Supplementary information

**Additional file 1.** Comic book.

**Additional file 2.** Instructions for text messaging.

**Additional file 3.** Instructions for taking your BP.

**Additional file 4.** Statistical analysis plan.

**Additional file 5.** Recruitment material.

**Additional file 6.** Consent form.

## Data Availability

As noted above, individual patient level data will be available through the University of Michigan institutional archive after de-identification.

## Appendix

### Informed consent materials {32}

The recruitment materials (Additional file 5) and consent form (Additional file 6) are available as supplementary material.

## Abbreviations

AE: Adverse event
BP: Blood pressure
CBPR: Community-based participatory research
CTCEA: Common Terminology Criteria for Adverse Events
DBP: Diastolic blood pressure
ED: Emergency Department
EHR: Electronic health record
FQHC: Federally Qualified Health Center
ICH: International Conference on Harmonization
IMM: Independent medical monitor
IRB: Institutional Review Board
NIMHD: National Institute on Minority Health and Disparities
PCP: Primary care provider
PI: Principal investigator
RCT: Randomized controlled trial
REDCap: Research Electronic Data Capture
SAE: Serious adverse event
SAP: System application and product
SBP: Systolic blood pressure
