# Supplementary material for: Reach out behavioral intervention for hypertension initiated in the emergency department connecting multiple health systems: study protocol for a randomized control trial
Source: Trials. 2020 Jun 3;21:456. doi: 10.1186/s13063-020-04340-z (PMC7268693; doi:10.1186/s13063-020-04340-z)
Supplement: Supplementary file 1 — Additional file 1. Comic book. [file 13063_2020_4340_MOESM1_ESM.pdf]

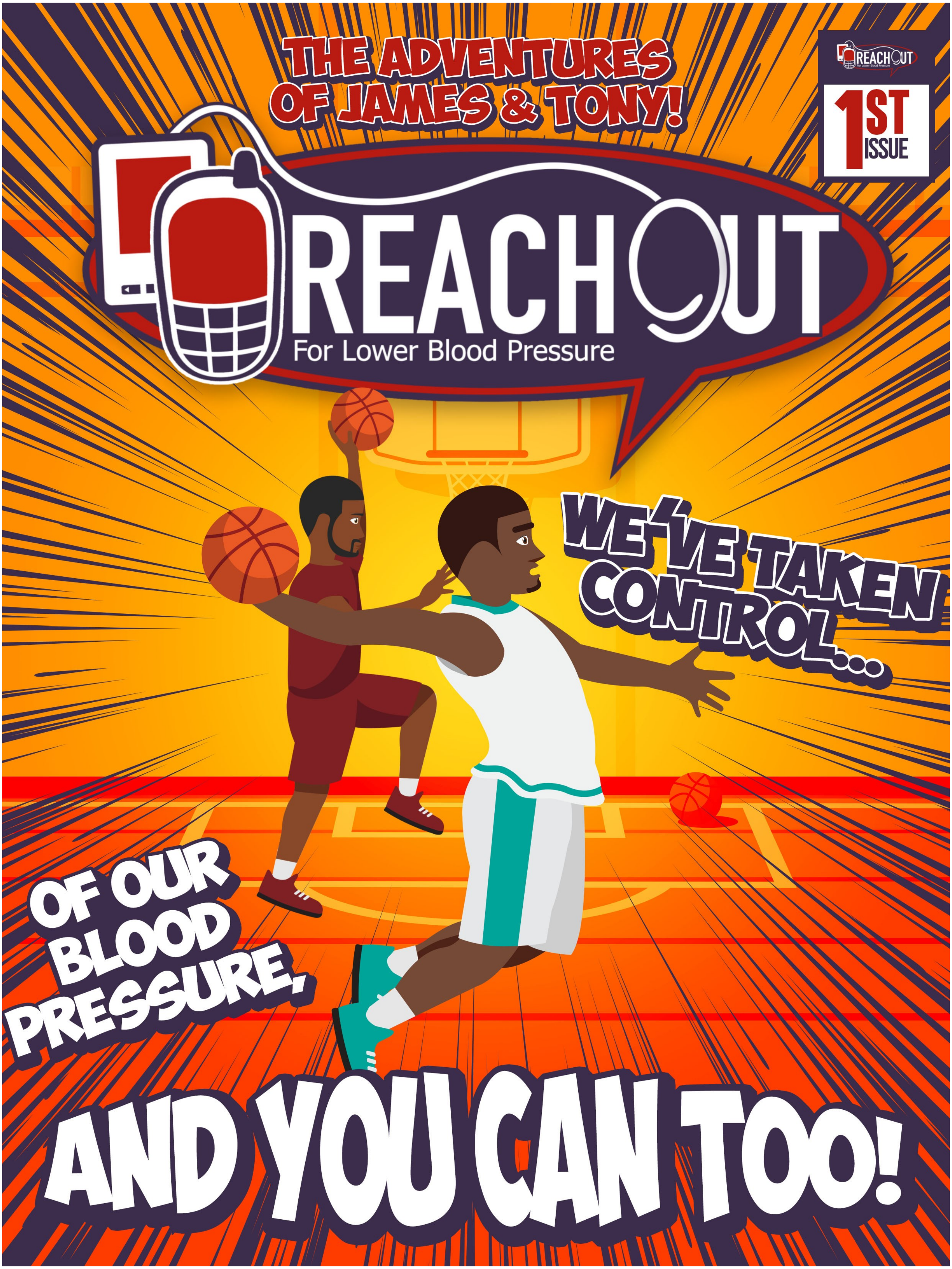

THE ADVENTURES OF JAMES & TONY!
REACHOUT
For Lower Blood Pressure
REACHOUT
1ST ISSUE
WE'VE TAKEN CONTROL...
OF OUR BLOOD PRESSURE,
AND YOU CAN TOO!

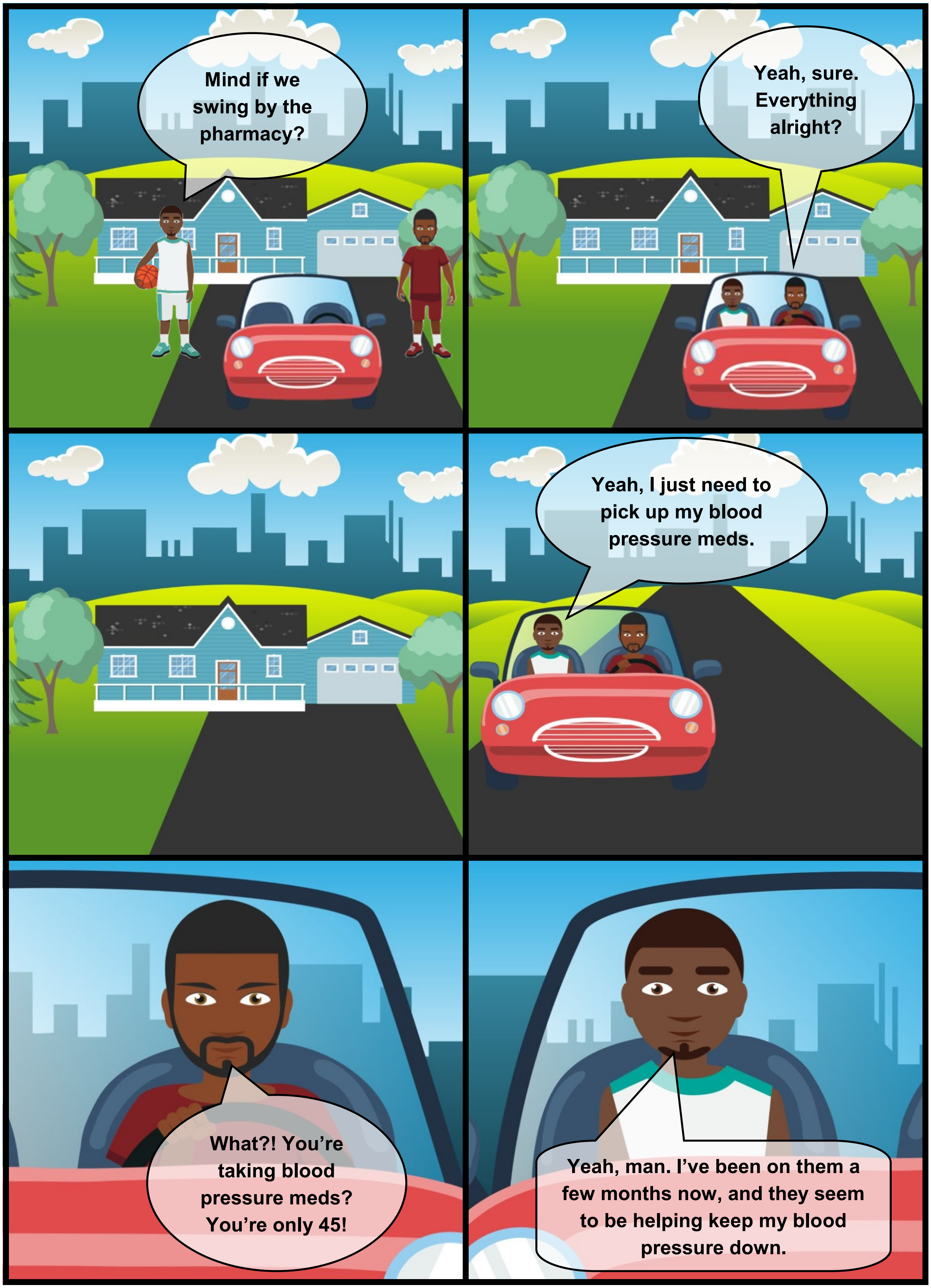
Mind if we swing by the pharmacy?
Yeah, sure. Everything alright?
Yeah, I just need to pick up my blood pressure meds.
What?! You're taking blood pressure meds? You're only 45!
Yeah, man. I've been on them a few months now, and they seem to be helping keep my blood pressure down.

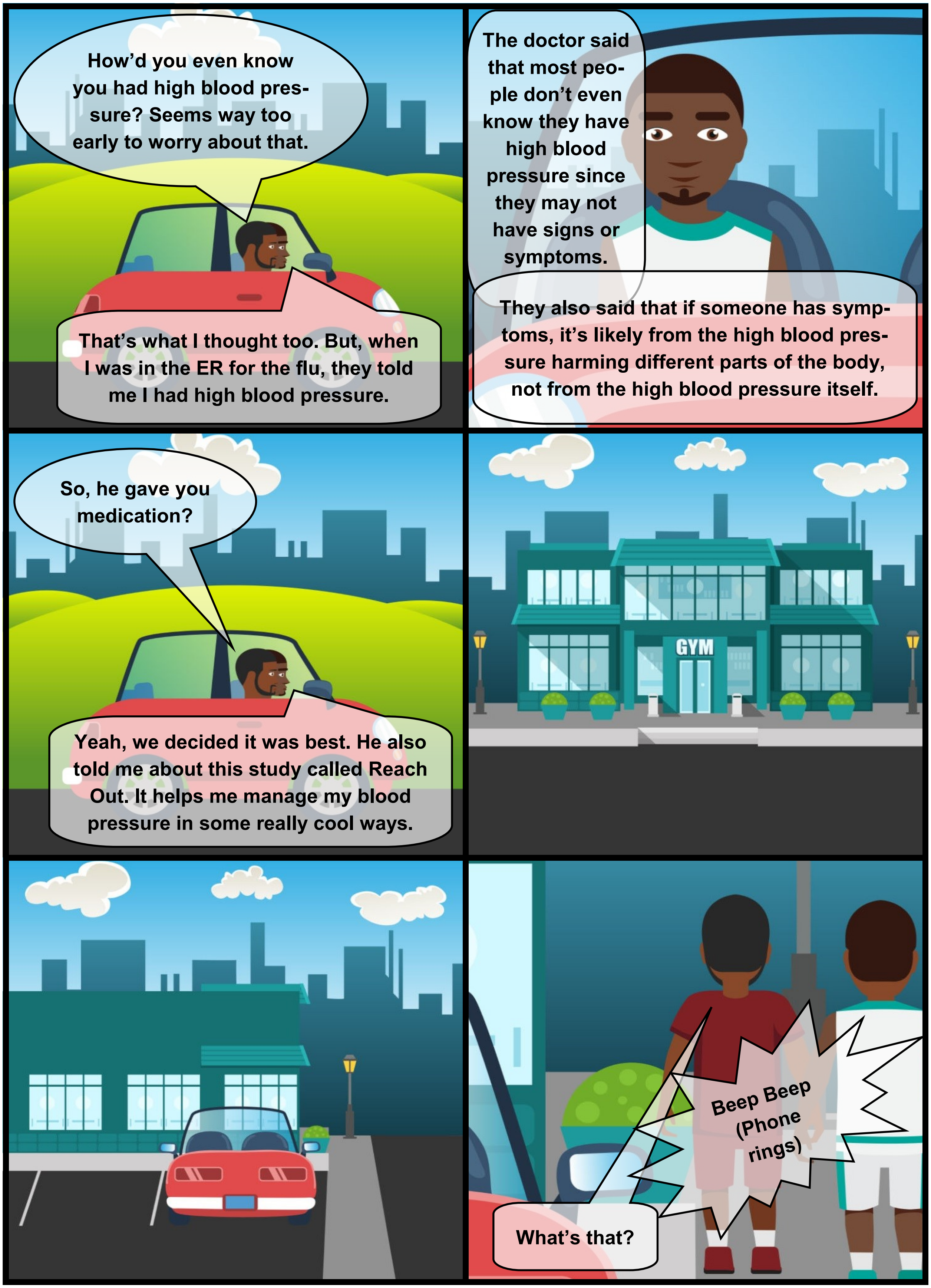
How'd you even know you had high blood pressure? Seems way too early to worry about that.
That's what I thought too. But, when I was in the ER for the flu, they told me I had high blood pressure.
The doctor said that most people don't even know they have high blood pressure since they may not have signs or symptoms.
They also said that if someone has symptoms, it's likely from the high blood pressure harming different parts of the body, not from the high blood pressure itself.
So, he gave you medication?
Yeah, we decided it was best. He also told me about this study called Reach Out. It helps me manage my blood pressure in some really cool ways.
GYM
Beep Beep (Phone rings)
What's that?

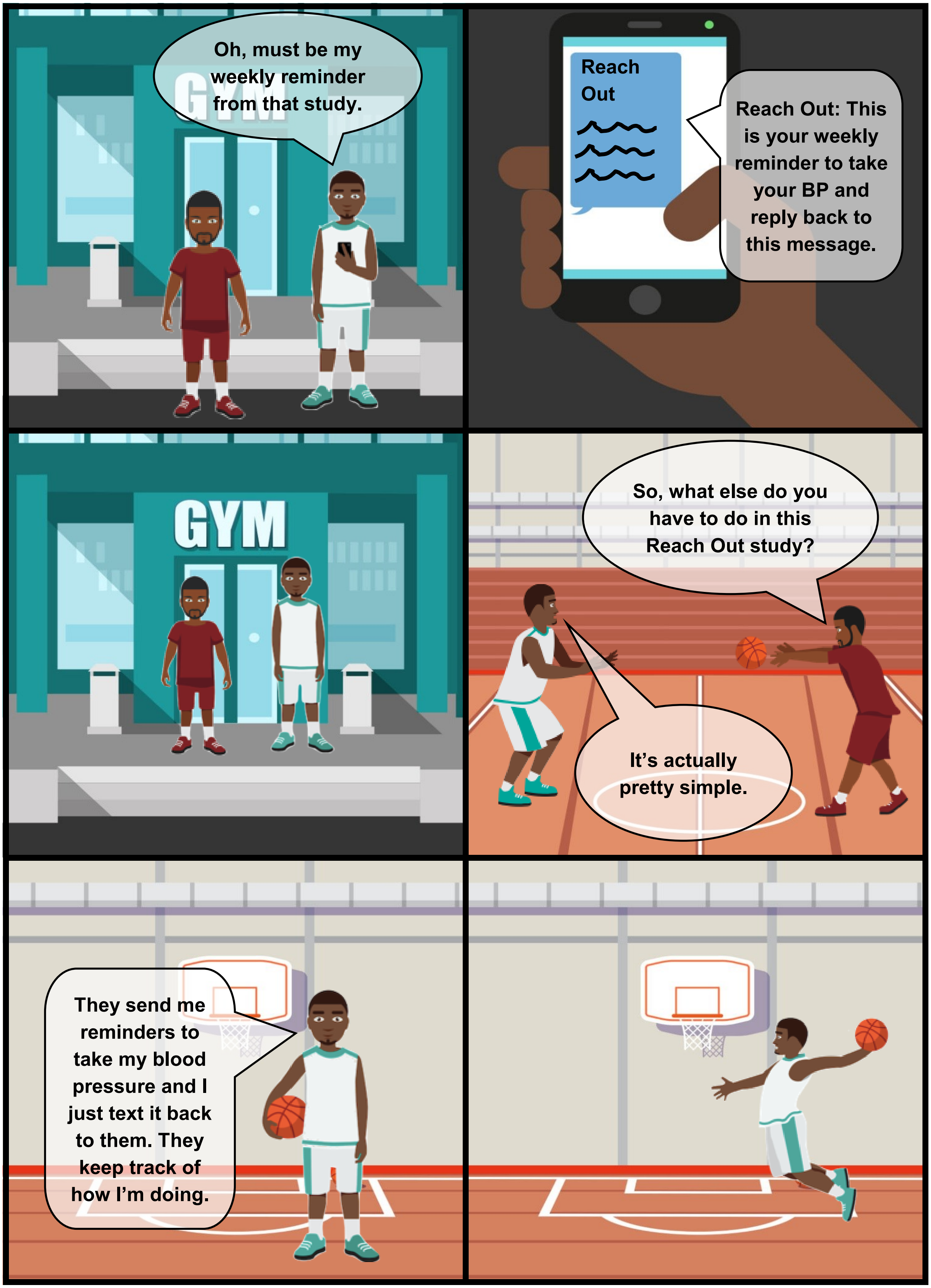
Oh, must be my weekly reminder from that study.
Reach Out
Reach Out: This is your weekly reminder to take your BP and reply back to this message.
GYM
So, what else do you have to do in this Reach Out study?
It's actually pretty simple.
They send me reminders to take my blood pressure and I just text it back to them. They keep track of how I'm doing.

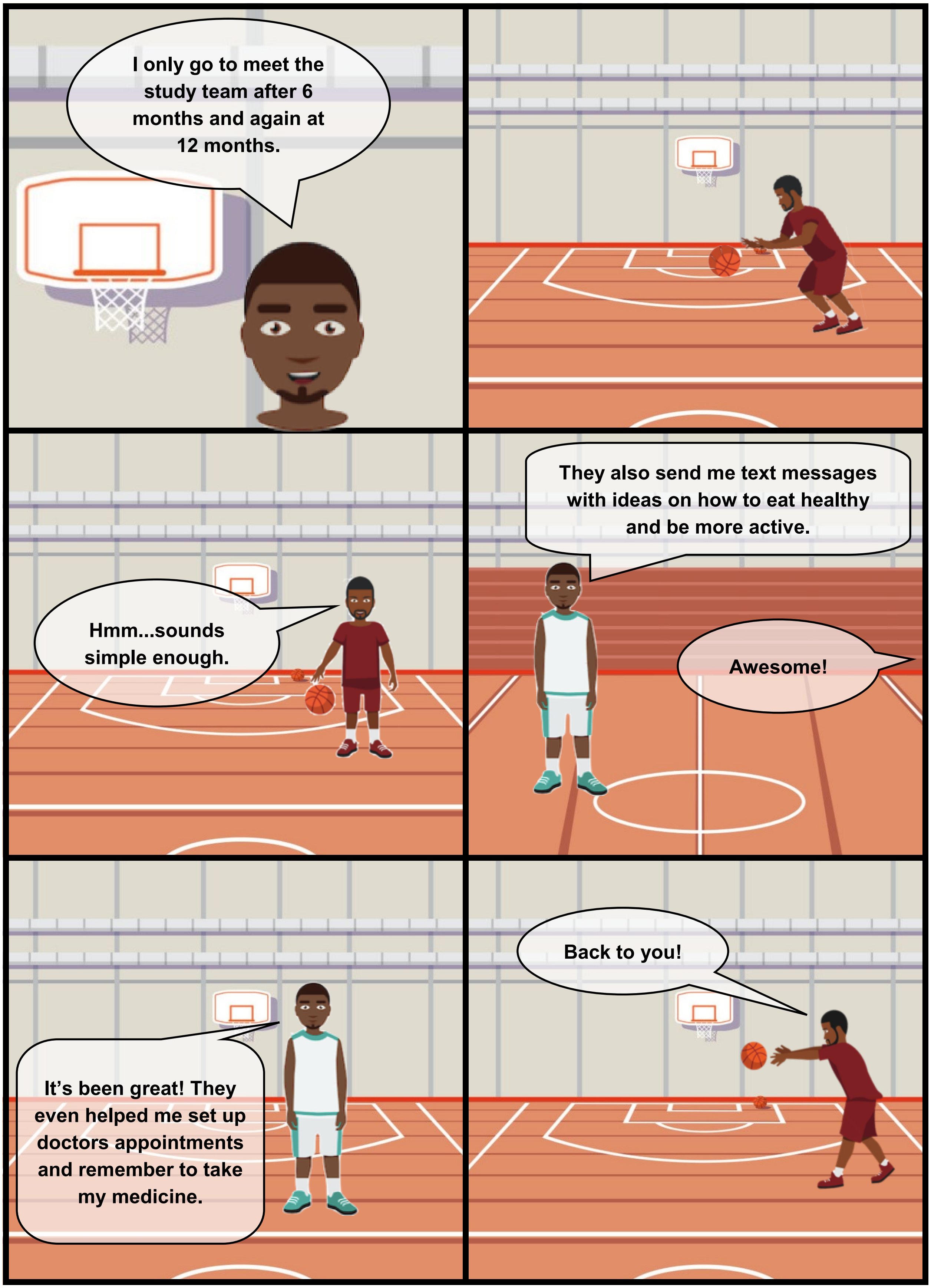
I only go to meet the study team after 6 months and again at 12 months.
Hmm...sounds simple enough.
They also send me text messages with ideas on how to eat healthy and be more active.
Awesome!
It's been great! They even helped me set up doctors appointments and remember to take my medicine.
Back to you!

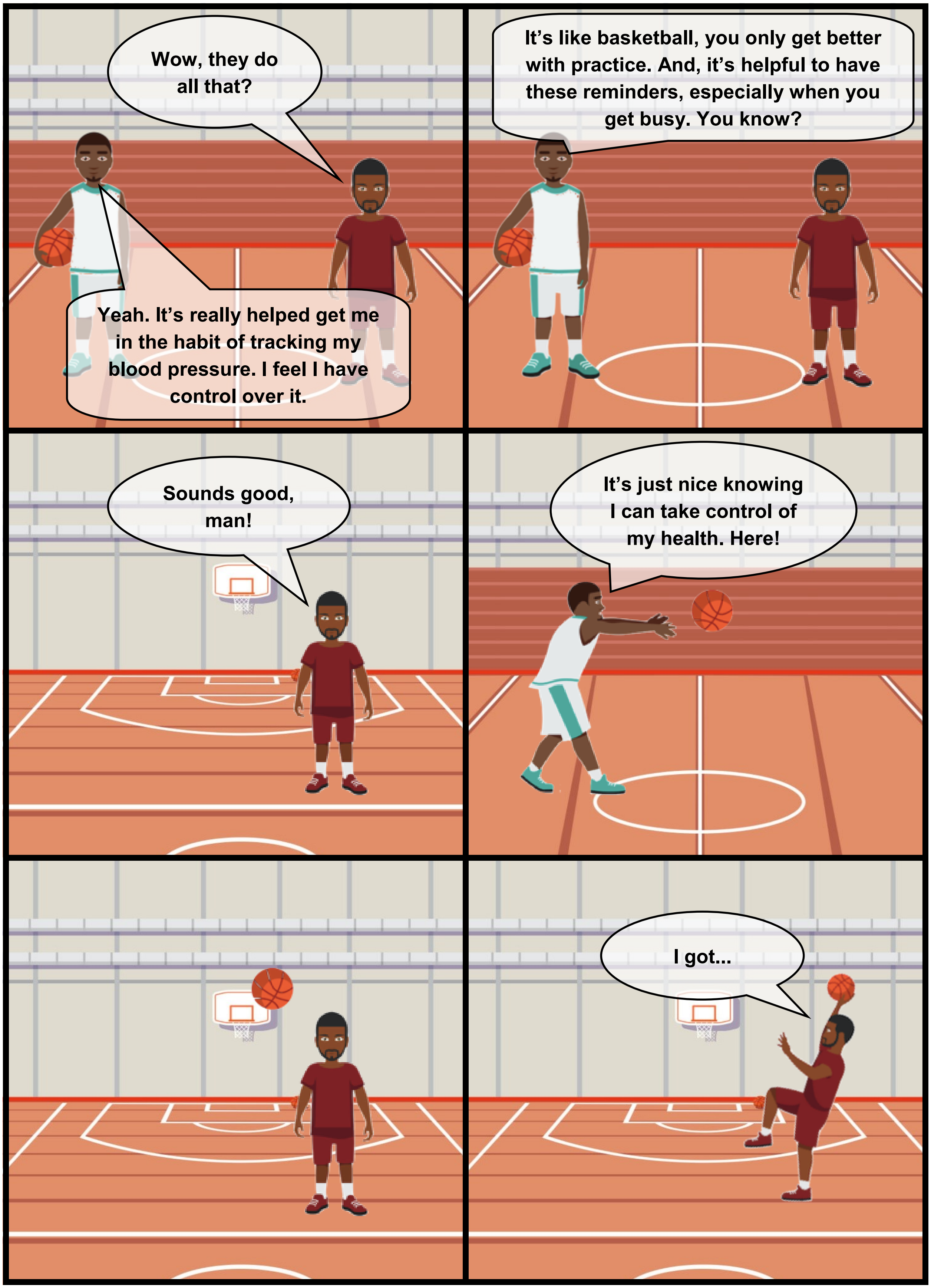
Wow, they do all that?
Yeah. It's really helped get me in the habit of tracking my blood pressure. I feel I have control over it.
It's like basketball, you only get better with practice. And, it's helpful to have these reminders, especially when you get busy. You know?
Sounds good, man!
It's just nice knowing I can take control of my health. Here!
I got...

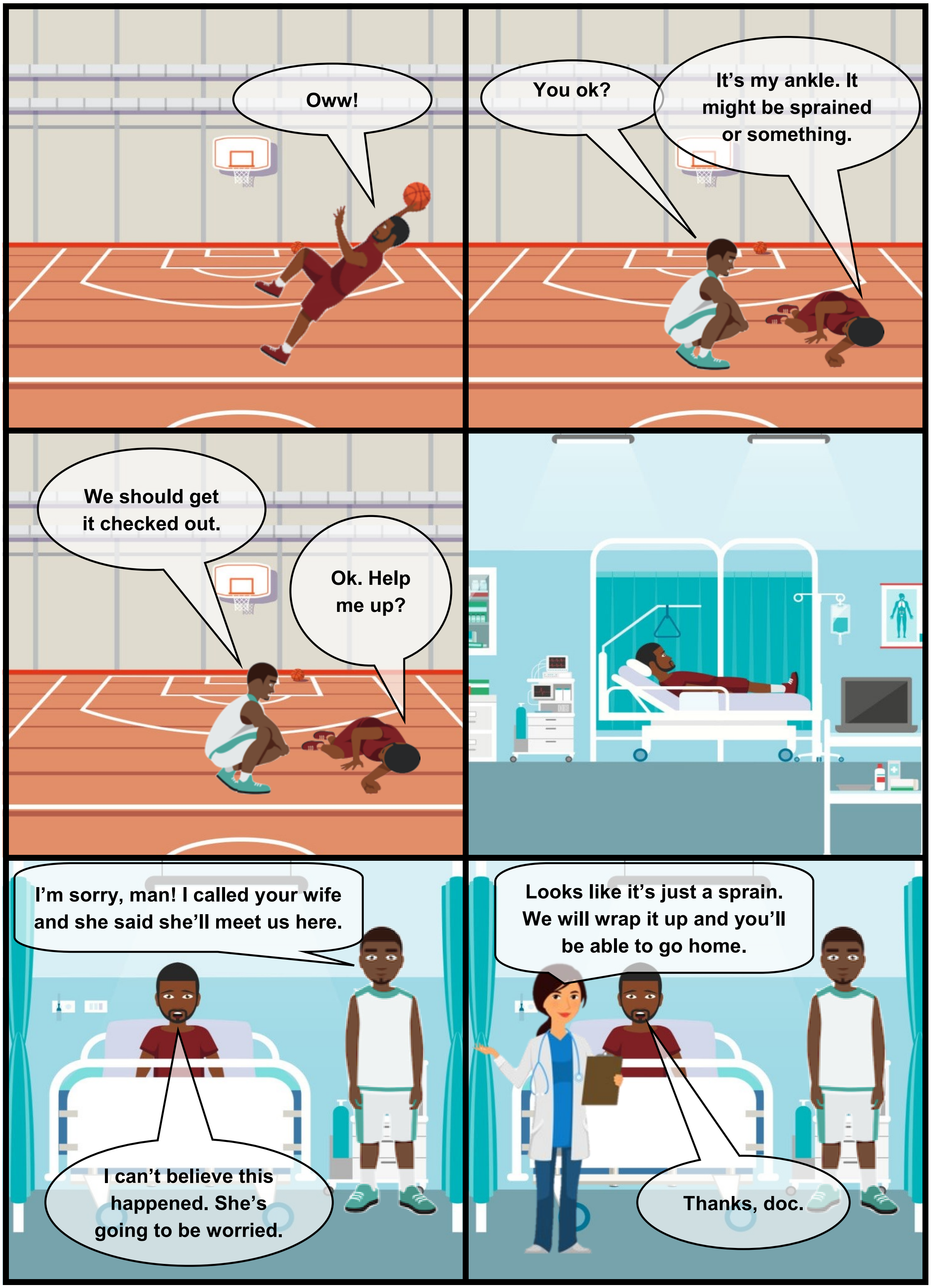
Oww!
You ok?
It's my ankle. It might be sprained or something.
We should get it checked out.
Ok. Help me up?
I'm sorry, man! I called your wife and she said she'll meet us here.
I can't believe this happened. She's going to be worried.
Looks like it's just a sprain. We will wrap it up and you'll be able to go home.
Thanks, doc.

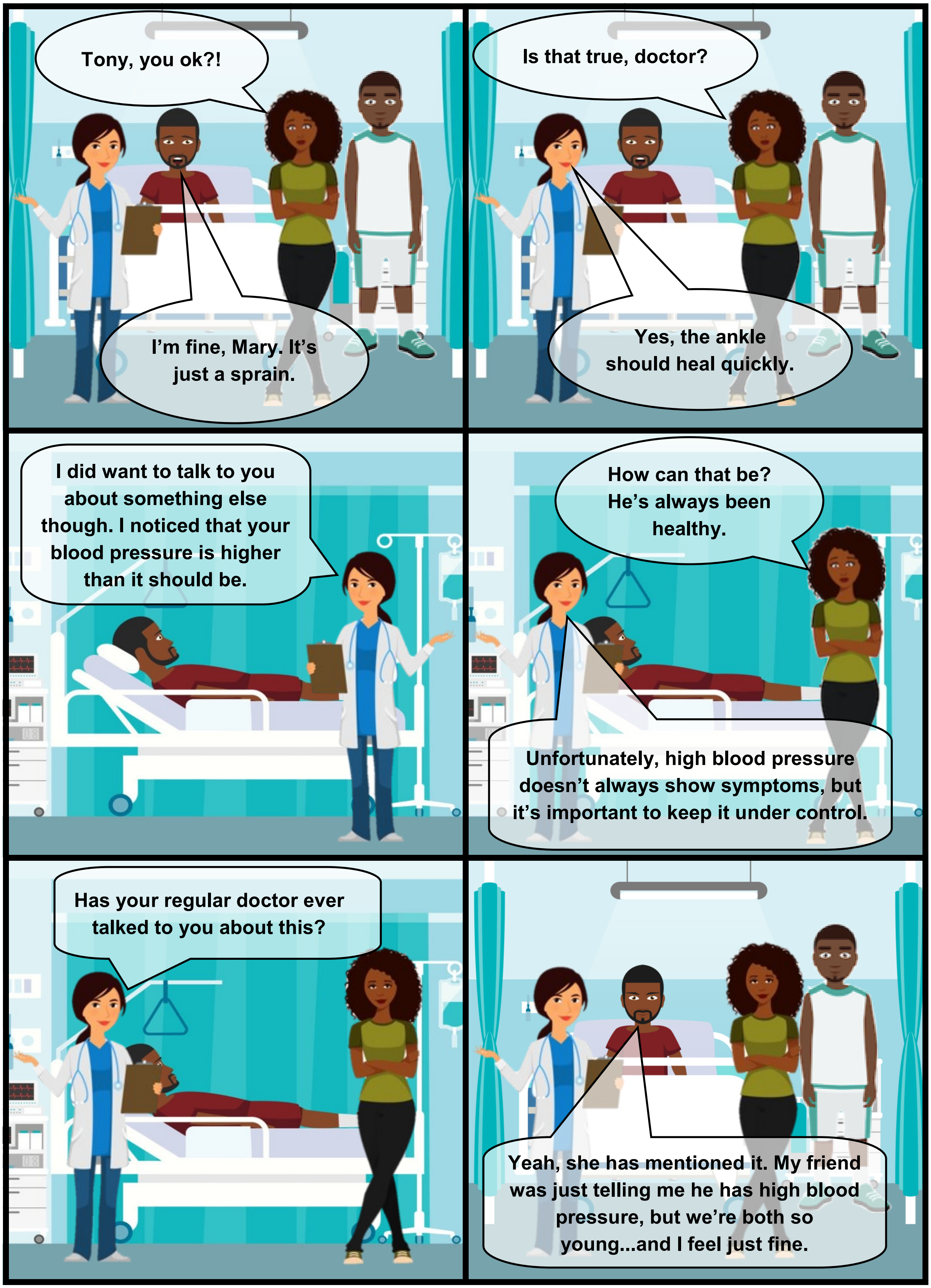
Tony, you ok?!
I'm fine, Mary. It's just a sprain.
Is that true, doctor?
Yes, the ankle should heal quickly.
I did want to talk to you about something else though. I noticed that your blood pressure is higher than it should be.
How can that be? He's always been healthy.
Unfortunately, high blood pressure doesn't always show symptoms, but it's important to keep it under control.
Has your regular doctor ever talked to you about this?
Yeah, she has mentioned it. My friend was just telling me he has high blood pressure, but we're both so young...and I feel just fine.

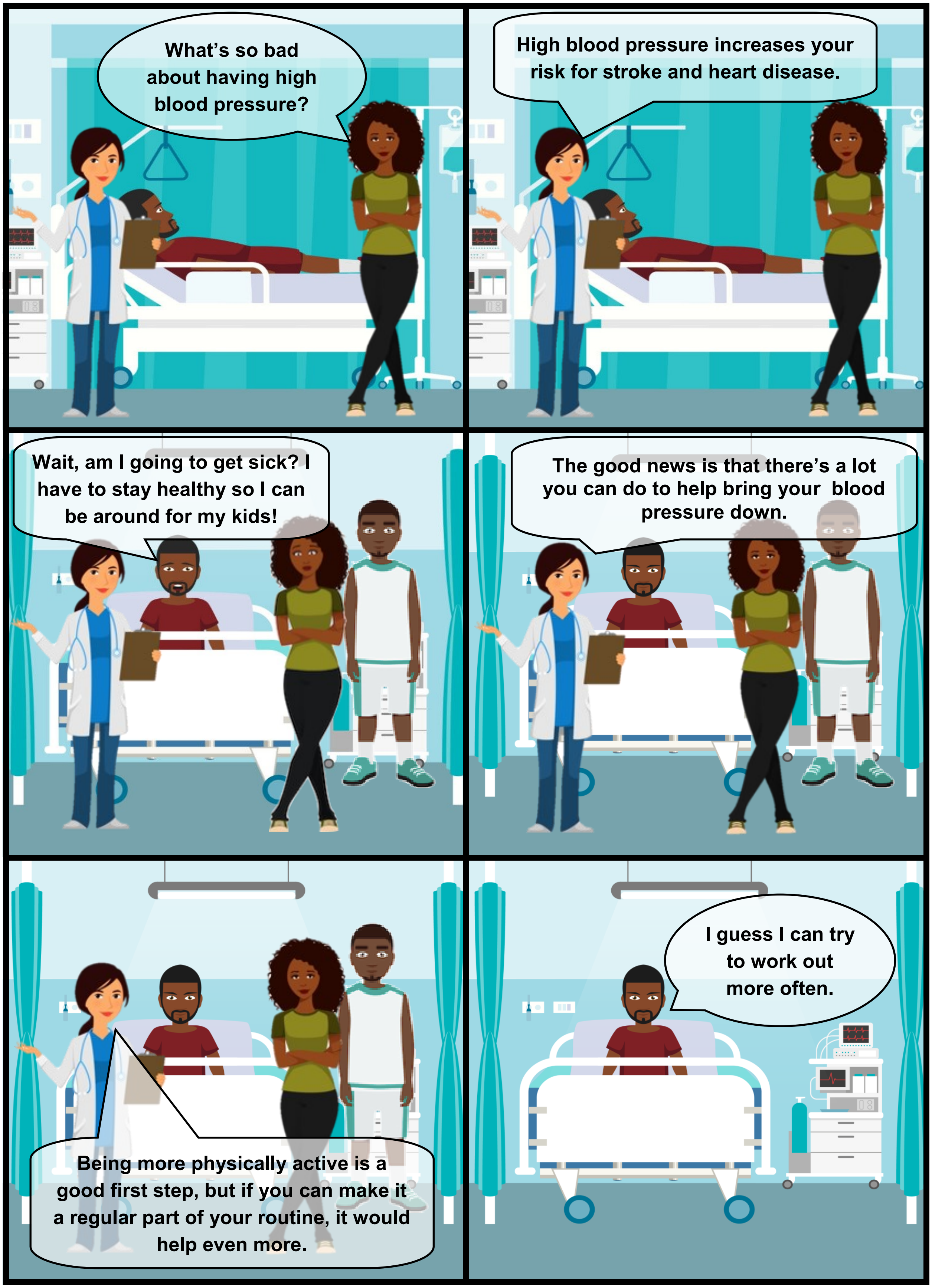
What's so bad about having high blood pressure?
High blood pressure increases your risk for stroke and heart disease.
Wait, am I going to get sick? I have to stay healthy so I can be around for my kids!
The good news is that there's a lot you can do to help bring your blood pressure down.
Being more physically active is a good first step, but if you can make it a regular part of your routine, it would help even more.
I guess I can try to work out more often.

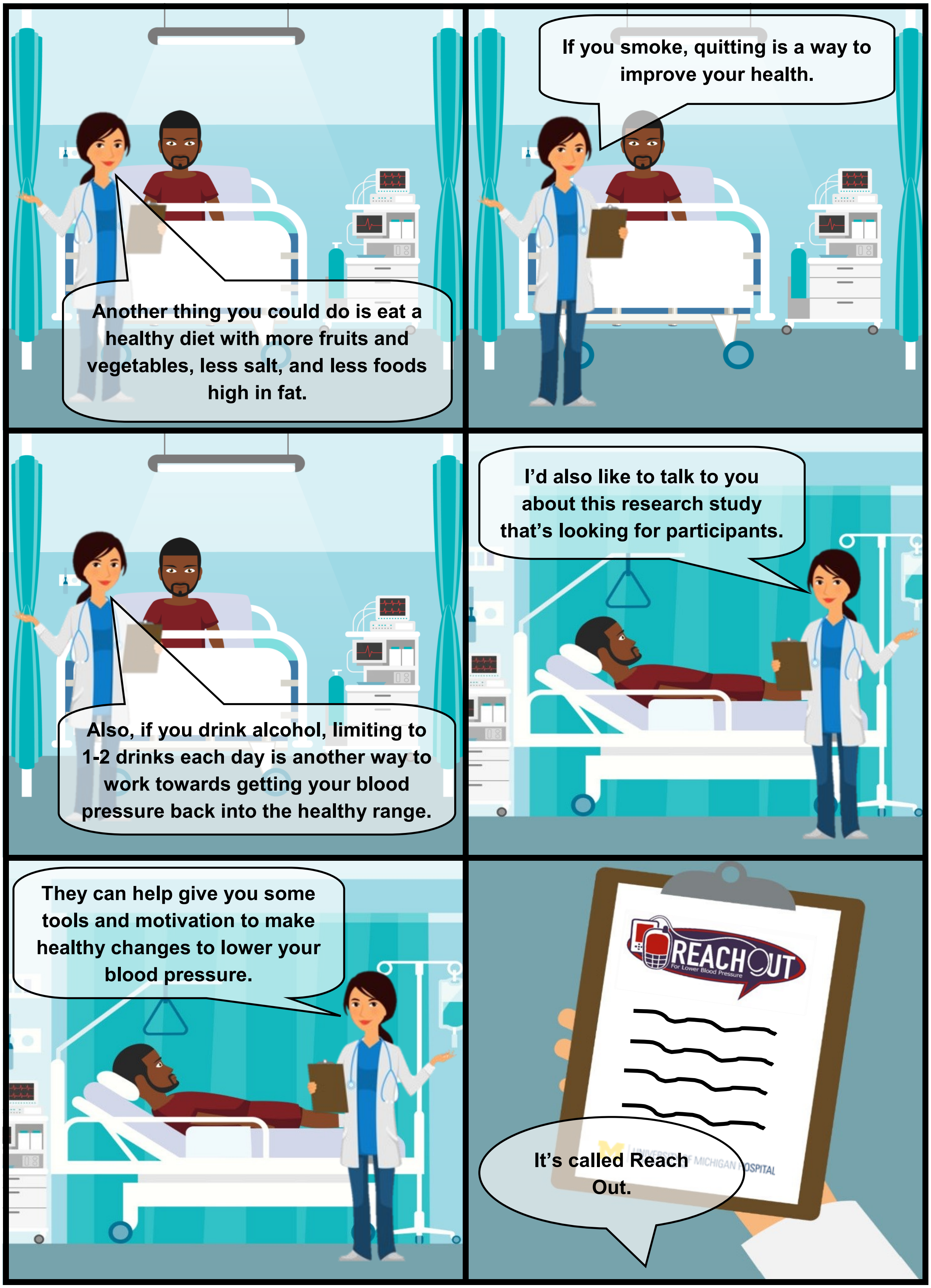
Another thing you could do is eat a healthy diet with more fruits and vegetables, less salt, and less foods high in fat.
If you smoke, quitting is a way to improve your health.
Also, if you drink alcohol, limiting to 1-2 drinks each day is another way to work towards getting your blood pressure back into the healthy range.
I'd also like to talk to you about this research study that's looking for participants.
They can help give you some tools and motivation to make healthy changes to lower your blood pressure.
REACH OUT
It's called Reach Out.

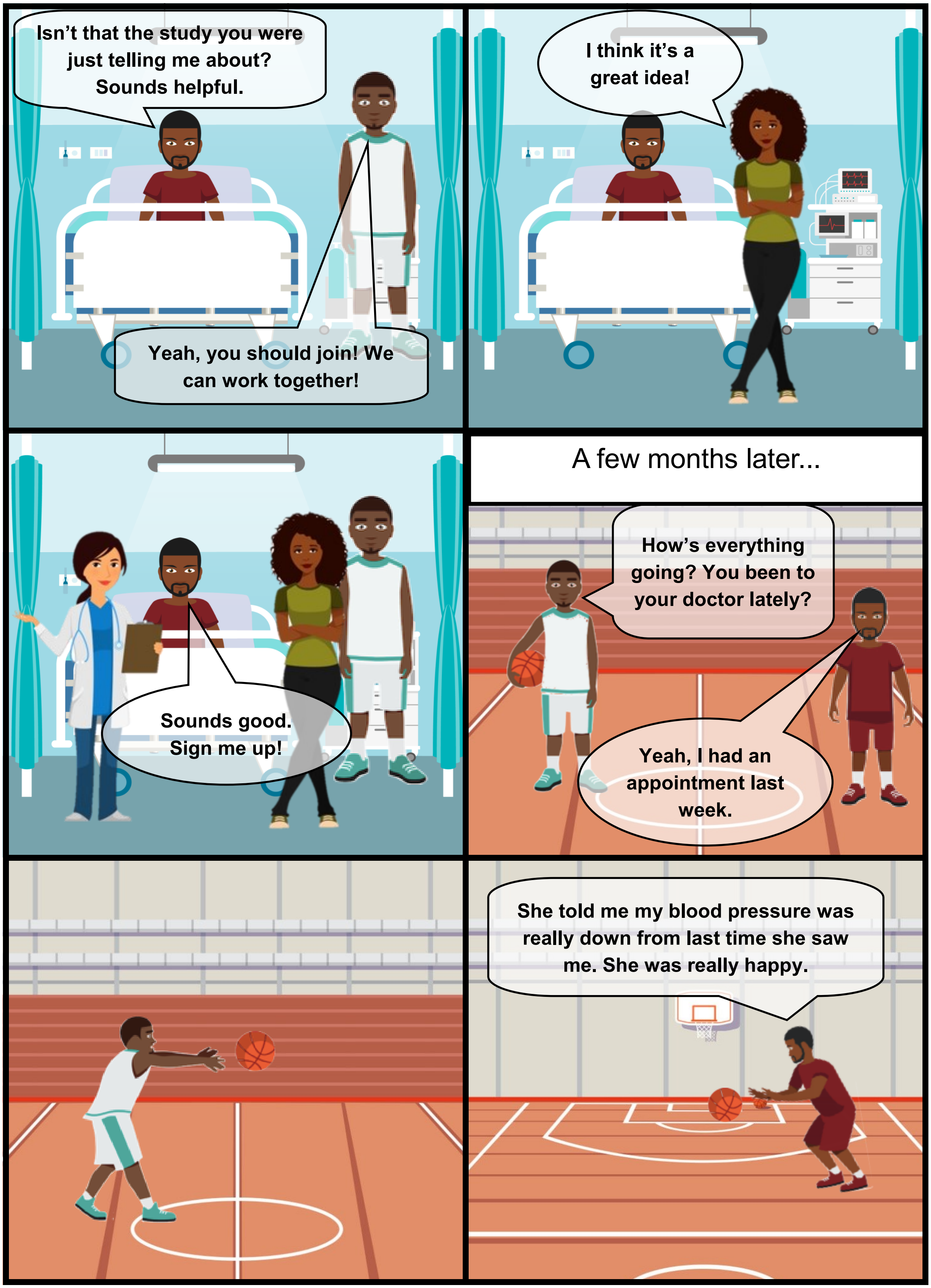
Isn't that the study you were just telling me about? Sounds helpful.
Yeah, you should join! We can work together!
I think it's a great idea!
Sounds good. Sign me up!
A few months later...
How's everything going? You been to your doctor lately?
Yeah, I had an appointment last week.
She told me my blood pressure was really down from last time she saw me. She was really happy.

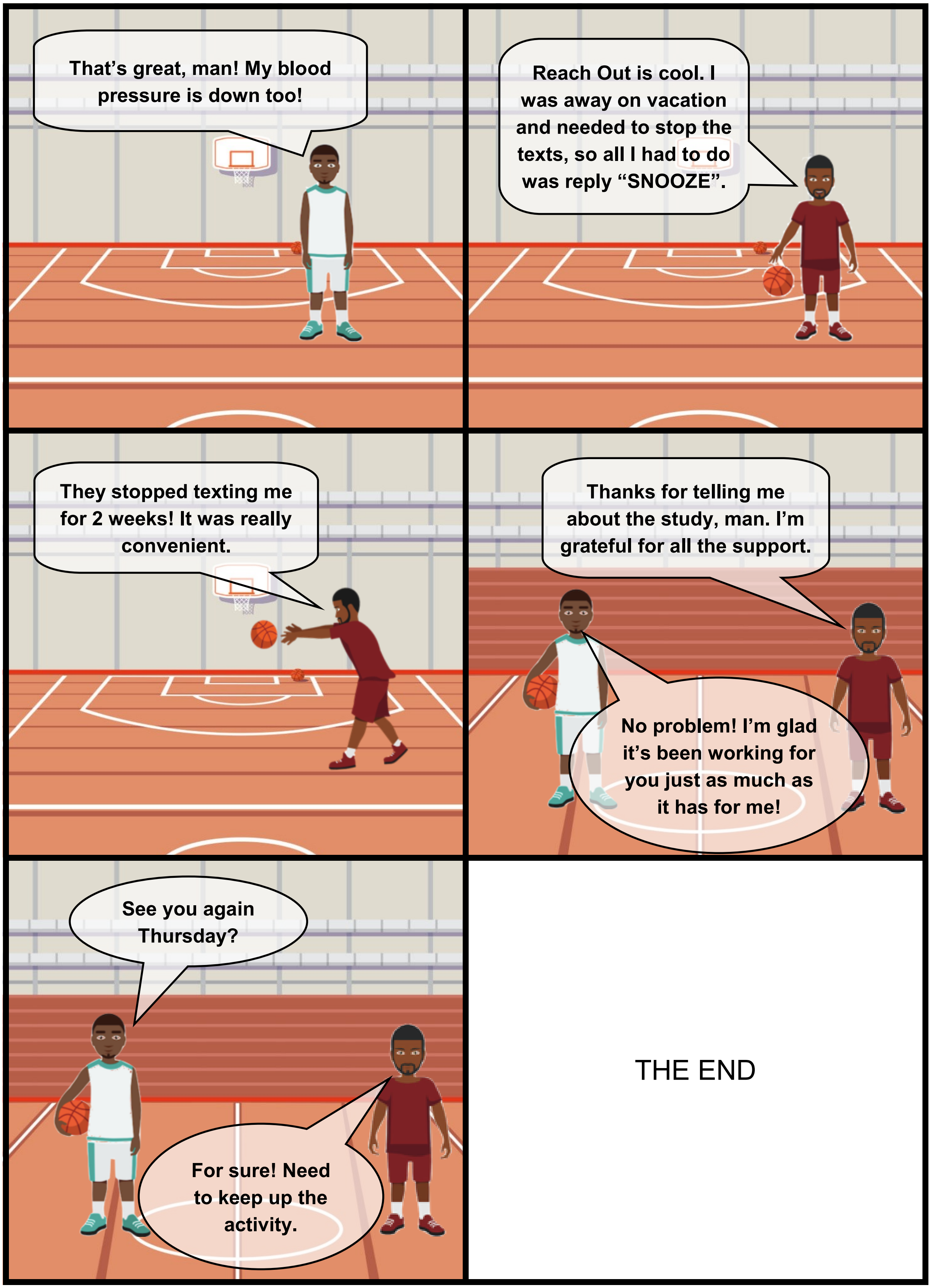
That's great, man! My blood pressure is down too!
Reach Out is cool. I was away on vacation and needed to stop the texts, so all I had to do was reply "SNOOZE".
They stopped texting me for 2 weeks! It was really convenient.
Thanks for telling me about the study, man. I'm grateful for all the support.
No problem! I'm glad it's been working for you just as much as it has for me!
See you again Thursday?
For sure! Need to keep up the activity.
THE END
