# Supplementary material for: Reach out behavioral intervention for hypertension initiated in the emergency department connecting multiple health systems: study protocol for a randomized control trial
Source: Trials. 2020 Jun 3;21:456. doi: 10.1186/s13063-020-04340-z (PMC7268693; doi:10.1186/s13063-020-04340-z)
Supplement: Supplementary file 2 — Additional file 2. Instructions for text messaging. [file 13063_2020_4340_MOESM2_ESM.pdf]

Instructions for Text Messages

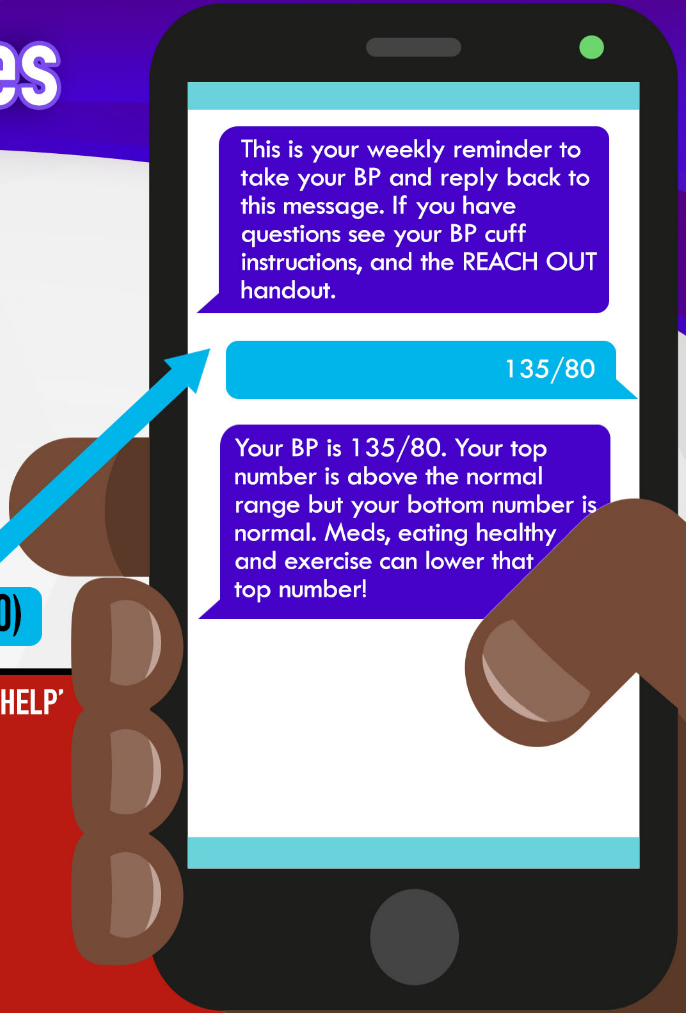

You will be getting text message reminders
to take your blood pressure (BP).
To make sure you are able to receive and send messages:

1. When you get the text from REACH OUT to take your BP, follow the instruction sheet for taking your BP at home
2. Text your BP to "REACH OUT" as xxx/xxx (for example: 135/80)

If you want to know all the changes you can make to your text messages, just text 'HELP'

You can take a break from getting text messages. Just text 'SNOOZE'

- After a 2-week break, you will start getting text messages again.

You can change how often you get text messages to remind you to take your BP.

- Text 'DAILY' to get text message reminders every day.
- Text 'WEEKLY' to get text message reminders once a week.

If you would like us to send a graph that tracks your BP readings, text 'GRAPH'

You can stop participating in the REACH OUT project at any time. Just text 'END'

Things to know before you decide to 'END':

- You won't receive any more text message reminders to take your blood pressure,
- We'll only contact you for follow-up at 6 months and 12 months.

Important: The text messages are not read by medical providers, so never text medical questions.
If you are having an emergency, please call 911

Abbreviations we may use:
BP = Blood Pressure
ED = Emergency Department
Dr = Doctor
Meds = medications

REACH OUT
For Lower Blood Pressure

Have questions about REACH OUT study? Call or text the REACH OUT staff at (810) 337-8399
