# Supplementary material for: Reach out behavioral intervention for hypertension initiated in the emergency department connecting multiple health systems: study protocol for a randomized control trial
Source: Trials. 2020 Jun 3;21:456. doi: 10.1186/s13063-020-04340-z (PMC7268693; doi:10.1186/s13063-020-04340-z)
Supplement: Supplementary file 3 — Additional file 3. Instructions for taking your BP. [file 13063_2020_4340_MOESM3_ESM.pdf]

Instructions for Taking Your Blood Pressure (BP) at Home

It is helpful to check your blood pressure throughout the week because your BP can go up and down. You should try to take your BP a total of 4 times each day:

- Take your BP 2 times in the morning, before you eat breakfast & before taking your medications for the day.
- Take your BP again 2 times in the evening, an hour before eating dinner.

1 Don't drink anything (coffee, pop, or alcohol), smoke, or workout within 1 hour of taking your BP.

2 Sit down and rest for at least 5 minutes before taking your BP. Sit calmly and don't talk.

3 Place your left elbow on a table so that the monitor will be at the same level as your heart. Keep your feet flat on the floor and don't cross your legs

4 Wrap the cuff securely around your left wrist. Push the power button to turn the machine on. The cuff will automatically inflate and measurement will start.

5 Wait 1 minute as the cuff automatically deflates, then push the power button again. You should have taken your BP a total of 2 times.

6 Reply to your REACH OUT text message with your first BP. You can see on the screen the BP was 120/80.

REACH OUT For Lower Blood Pressure

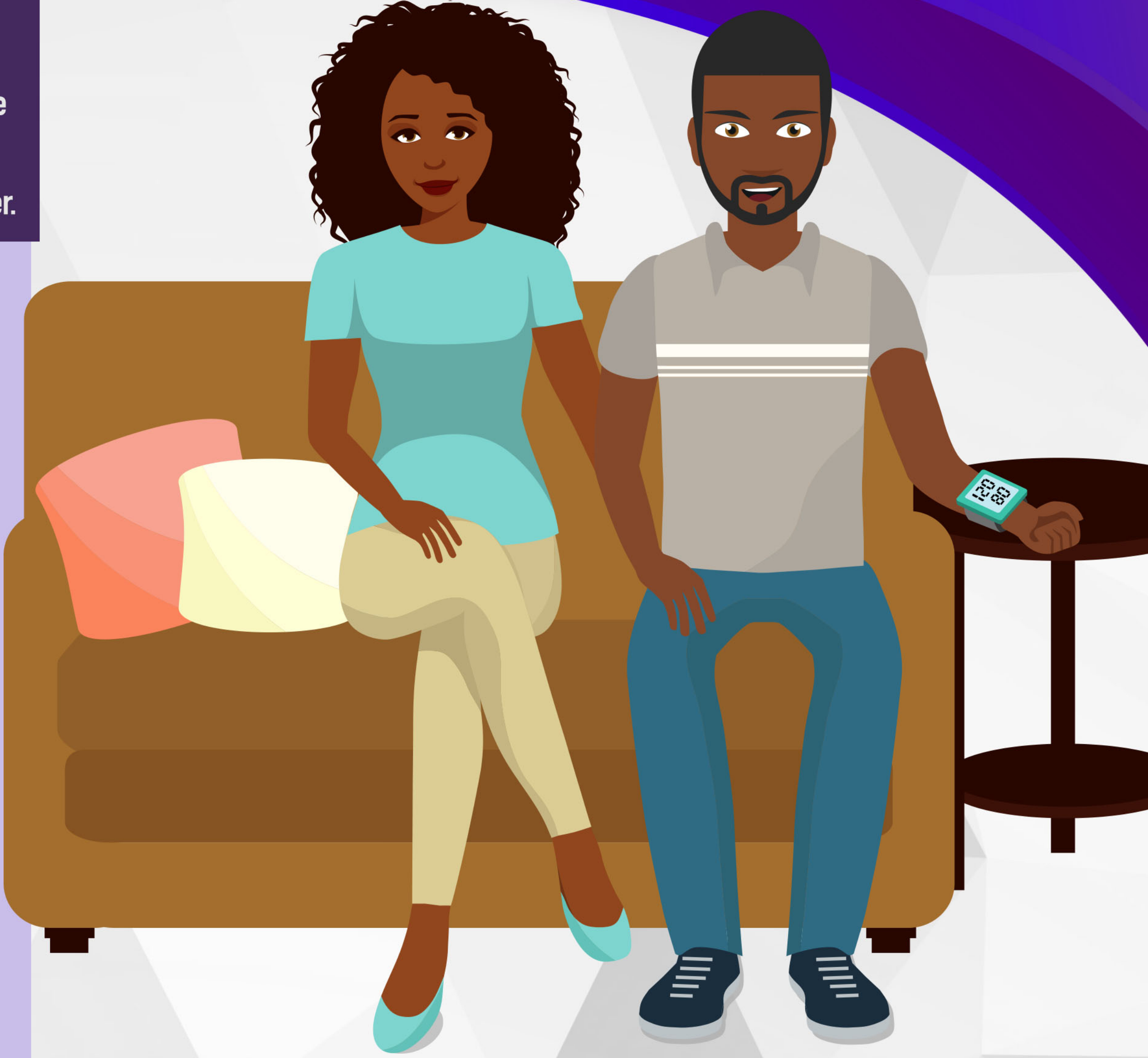

Important: The text messages are not read by medical providers, so never text medical questions. If you are having an emergency, please call 911

Have questions about REACH OUT study? Call or text the REACH OUT staff at (810) 337-8399
