# Supplementary material for: Reach out behavioral intervention for hypertension initiated in the emergency department connecting multiple health systems: study protocol for a randomized control trial
Source: Trials. 2020 Jun 3;21:456. doi: 10.1186/s13063-020-04340-z (PMC7268693; doi:10.1186/s13063-020-04340-z)
Supplement: Supplementary file 4 — Additional file 4. Statistical analysis plan. [file 13063_2020_4340_MOESM4_ESM.doc]

**Reach Out**

**Statistical Analysis Plan**

September 2018

### **Review of statistical design**

| Table 1: Reach Out ED Intervention Arms | | | |
| --- | --- | --- | --- |
| Arm | Healthy Behavior Text | Prompted BP self-monitoring frequency | Facilitated Primary Care appointment scheduling and transportation |
| 1 | No | Low | No |
| 2 | Yes | Low | No |
| 3 | No | High | No |
| 4 | Yes | High | No |
| 5 | No | Low | Yes |
| 6 | Yes | Low | Yes |
| 7 | No | High | Yes |
| 8 | Yes | High | Yes |

Reach Out is a health system focused, multicomponent, multiphase optimization strategy (MOST), health theory based, mobile health behavioral intervention to reduce BP among hypertensive patients evaluated in a safety net ED. This trial will take place in Flint, Michigan, an urban, under-resourced, predominately African American community with which the researchers have long-standing partnerships. Reach Out consists of three components, each with two levels: 1) healthy behavior text messaging (yes vs. no), 2) prompted home BP self-monitoring (weekly vs. daily), and 3) facilitated primary care provider appointment scheduling and transportation (yes vs. no).

Subjects will be block randomized into one of the eight experimental arms (Table 1) and followed for 12 months. Importantly, the intervention components may have important associations with subjects’ baseline characteristics. We are most interested in three baseline characteristics: age, sex, and antihypertensive use. Thus, to ensure scientific rigor, in randomization we will reduce imbalance within our treatment arms by stratifying randomization with these variables: 1) age (<64, ≥65); 2) sex, as reported by participant; 3) taking an antihypertensive within the last 6 months (yes vs. no).

Our overarching objective is to demonstrate that an ED initiated, multicomponent, health system intervention can meaningfully reduce BP in a traditionally underserved population.

**Primary Objective**: Estimate the expected change in 12-month SBP reduction associated with each level of the three interventions and determine the combination of elements that appears most promising for study in a future larger scale trial.

**Primary Endpoint**: The primary endpoint is the change in systolic BP from baseline to 12 months.

**Secondary Endpoints**: Time from ED visit to arrival at first primary care visit (in days); Attendance at two or more primary care visits within 12 months of randomization

**Sample Size**: We plan to enroll approximately 960 patients into the eligibility phase. From this group, we estimate that 480 subjects will report qualifying BPs and will be randomized to one of the eight intervention arms. We anticipate 240 subjects will fully complete the 12 month, in person follow up visit. For 80% power to detect the smallest main expected effect of 0.35 based on the Collins method of a 3-factor experiment analyzed using a second order model (i.e. main effects and 2-way interactions), we require 196 subjects. Assuming 480 randomized subjects and allowing an additional 50% loss to follow up after the eligibility phase, we expect at least 240 subjects, exceeding 80% power with an alpha level of 0.10 (inflated to reduce likelihood of dismissing active components with modest BP reducing effect) – we used the FactorialPowerPlan SAS macro to calculate power. For the secondary analyses, considering an alpha of 0.05 and a baseline proportion attending 2 or more primary care provider visits within one year of 40%, with a total sample size of 240 evenly distributed between the active appointment scheduling and passive appointment, we have 87% power to detect an increase to 60% attending 2 or more visits. The time to event analysis (considering an alpha of 0.05, power of 99%, and 240 subjects enrolled over 720 days, followed for 30 days) can detect a change from 20% of subjects in the non-facilitated group to 20.4% of subjects in the facilitated group.

**Analysis Plan**: The primary analysis (aim 1) will fit a linear regression model with the outcome of SBP change (baseline minus 12 months) and main effect-coded binary predictors of healthy behavior texts (yes vs. no), prompted BP self-monitoring frequency (high vs. low), and primary care provider visit scheduling and transportation (active vs. passive). Initial analyses will focus on the main effects. Additional analyses will include all the two-way interactions of the three intervention components (only considering interactions where at least one of the factors in the interaction demonstrates a sufficiently large main effect). As the goal of this exploratory trial is to find interventions and combinations with activity, we will use an alpha level of 0.10 for all main effects and 0.20 for interactions. We plan to include elements meeting that bar for statistical significance in the subsequent multicenter trial. If significant interactions exist (i.e. the combination of facilitated transportation and high frequency home BP monitoring), we plan to implement the combination of elements that has the highest expected reduction in SBP – assuming we achieve significance at the p=0.1 (main effects) or 0.2 (interaction) level for the components. In the event of an overall “null” trial, we would examine the expected change in SBP for arm 1, which would be represented by the intercept. If this was significantly greater than 0 at the p=0.1 level we would propose a very simple subsequent multi-center trial using only weekly prompted BP self- monitoring.

The main secondary analyses (aim 2) will use time-to event (Cox Proportional Hazards) and logistic regression. For the endpoint of interest, (either time to first primary care visit, or the binary variable indicating attendance at two or more primary care visits within 1 year of randomization), we will fit an adjusted regression model. The main predictor of interest for these models is assignment to the active primary care provider scheduling and transportation arm (50% of all subjects randomized in the trial), while adjusting for the assignment into other groups similar to aim 1 (healthy behavior text messages and prompted BP self-monitoring).

**2. Analysis Plan**

**2.1 Preliminary evaluation of data:**

***Purpose:*** To identify issues that will affect our approach to the primary analysis. Specifically:

to summarize the amount of missing data for key variables and to examine the distributional characteristics of key variables in order to detect potential data entry/reduction errors

***Methods:***  All intervention groups will be combined. Any variable that requires creation from collected data will be coded and created for examination. Continuous variables will be summarized by minimum, maximum, arithmetic mean, median, and standard deviation. Histograms and boxplots will be used to assess distributional characteristics of baseline and follow-up variables. The change in systolic BP from baseline to 12 months will be summarized and plotted to assess distributional characteristics. Categorical variables will be tabulated with frequencies and percentages. The number and percentage of missing observations for all variables will be reported for each variable for the entire sample. Missingness for the primary and secondary outcome variables will be assessed by intervention group to evaluate any differential missingness.

**2.2. Description of study process**

***Purpose:*** To compare how the study unfolded with the pre-trial plan to determine if changes in the pre-trial analysis plans are warranted.

***Methods/Approach:***

CONSORT flow figure

Number screened; number enrolled

Subjects randomized (number to each group)

Retention by group

Final completion rates

Number of subjects in analysis data sets

**2.3. Description of participants**

***Purpose:*** To describe the patient population by intervention group.

***Methods/Approach:*** Construct “table 1” describing the baseline characteristics of each intervention group including:

Demographic characteristics

Baseline medical history, Tobacco/Alcohol Information, Insurance Status, Transportation Status, Social Cognitive Theory Measures, medication history, medication adherence

Baseline values of primary and/or secondary outcome variables: BP, PCP Attendance

Means and standard deviations will describe continuous variables, frequencies and counts will describe categorical variables. Significance testing of baseline differences between groups will not be performed based on statistical and CONSORT recommendations.^1-2^

Intervention Exposure: Participants receiving daily BP monitoring or healthy behavior text messaging can choose to “snooze” (stop texts for 2 weeks) or de-escalate their treatment “weekly” (stop healthy behavior text messages, move to weekly BP monitoring). Participants with weekly BP monitoring can choose to “snooze” treatment (stop texts for 2 weeks) or escalate their treatment “daily” (daily BP monitoring no change to healthy behavior text messaging). The number and percentage of participants who “snooze” BP monitoring and healthy behavior texts, both daily and weekly, will be reported separately. The number and percentage of participants who choose to de-escalate from daily BP monitoring and healthy behavior texts will be reported separately as well the number and percentage who escalate their treatment. The proportion of all possible BP monitoring texts (either daily or weekly) completed for each subject will be calculated and the proportion and 95% confidence interval will be reported by intervention group.

**2.4. Primary analysis/results**

***Purpose:*** Primary results for the intention to treat (ITT) analysis.

***Methods/Approach:***

Intention to Treat Dataset: all subjects randomized into study and all data observed from each subject regardless of compliance and adherence with the assigned intervention or follow-up.

*Primary outcome:*  Change in systolic BP from baseline (median of three qualifying SBP from eligibility phase) to 12 months. Baseline minus 12 months.

*Primary analysis:* The primary analysis will fit a linear regression model with the outcome of SBP change and main effect-coded binary predictors of healthy behavior texts (yes vs. no), prompted BP self-monitoring frequency (high vs. low), and primary care provider visit scheduling and transportation (active vs. passive) and the stratification variables. Initial analyses will focus on the main effects. The outcome will be transformed (e.g., log) if necessary.

(BL BP- 12 mo BP) = β_0_+ β_1_HBtexts + β_2_HighBPmonitor + β_3_PCPfacilitated + β_4_female + β_5_Age>65 + β_6_AHT + β_7_Black

Where HBtexts= 1 for daily healthy behavior texts and 0 for none; HighBPmonitor=1 if daily BP monitoring and 0 for weekly; PCPfacilitated=1 for provider scheduling and transportation facilitated and 0 for not facilitated; female = 1 for self-reported female, 0 for male; Age>65=1 for Age≥65, 0 for 18-64; and AHT=1 for antihypertensives in the past 6 months, 0 for not; Black=1 for Black, 0 for Other

***Missing data:***

We will work to prevent missing data by the recruitment and retention strategies described elsewhere. The amount and patterns of missing data and its associations with other variables (in particular intervention category) will be explored so that an appropriate statistical method for analysis can be used. If the data is missing at random (missing outcomes can be predicted from other observed variables, especially recent self-reported blood pressures), which we believe to be the most likely, since we obtain text messaging-based BPs at least weekly, the repeated measurements within individuals will allow us to use multiple imputation to handle sporadic missing at random BP measurements. For multiple imputation, we will use R package mice to impute the data using multiple imputation by chained equations. Non-normal continuous missing variables will be transformed for normality and back-transformed for any calculations necessary (e.g., change in BP). The imputation model will include all variables in the analysis model, treatment interactions, as well as other covariates that predict the missing variable and whether the missing variable is missing. Thus, associations between baseline variables and missing variables will be assessed both for strength of association and functional form. We will use 100*fraction of incomplete cases number of imputations. Analyses will combine regression estimates using Rubin’s rules. Model checking will be performed on each imputed dataset through residual analysis to identify any issues with the imputation model or analysis model. Problems occurring in a small number of imputed datasets suggest a problem with the imputation dataset and problems occurring consistently across all imputed datasets suggest a problem with the analysis model.

In the case of non-ignorable missing (missing not at random) data, sensitivity analyses will be performed using pattern mixture or selection models to evaluate the robustness of our conclusions to a range of sensible conditions (for both the primary and additional analyses of the primary endpoint).

***Interpretation:***

Results will be reported as parameter estimates, 95% confidence intervals, and 2-sided p-values. As the goal of this exploratory trial is to find interventions and combinations with activity, we will use an alpha level of 0.10 for all main effects. Plots of mean BP levels over time will be generated for the 3 main effects and each intervention group.

**2.5. Secondary analyses**

***Purpose:***

1. To evaluate interactions of treatments in the change of BP over 12 months.
2. To evaluate the pattern of change of BP over time.
3. To evaluate time from ED visit to arrival at first primary care visit (in days).
4. To evaluate attendance at two or more primary care visits within 12 months of randomization.

***Methods/Approach:***  The following secondary analyses will be presented:

1. Other analysis approaches on the primary measurement:

Additional analyses will include all the two-way interactions of the three intervention components (only considering interactions where at least one of the factors in the interaction demonstrates a sufficiently large main effect) added to the primary model in Section 2.4. Interactions between treatments will be assessed at p=0.2.

Since BP levels are captured at least weekly, we will also model BP (captured by text and in person visits) over time using a mixed effects model to see the pattern of blood pressure levels over time. The outcome of this longitudinal model will be BP levels over time (transformed if necessary), where the number of BP levels per person can vary. The fixed effects of the model include: the 3 main effects binary coded treatment predictors and the 3 stratification variables as specified in the primary analysis model. A random slope for a continuous time variable measured in days from randomization and a random intercept to allow subject specific heterogeneity in baseline BP and BP trajectories over time. We will assume compound symmetry for the covariance matrix but assess the appropriateness of this assumption using AIC and BIC compared to unstructured and autoregressive structures. We will also assess the significance of two-way interactions of treatments (only where at least one of the factors demonstrates a sufficiently large main effect) in this model at the 0.2 level. Mixed effects models are robust to data missing at random.

As a sensitivity analysis, similar to a per protocol analysis, we will assess the effect of using group variables that may change from week to week as opposed to the given randomized treatment variable that stays fixed over the treatment period (intent to treat group). For example, if someone de-escalates from daily to weekly monitoring in a particular week, we can capture this in a longitudinal model by allowing their group variable to change from daily to weekly in that week. This will allow us to connect the BP measurement with the group assignment for that week, thus we will use all BP measurements. Otherwise, the mixed model construction will be the same as detailed in the paragraph above.

1. Other outcomes (secondary outcomes):
   - To evaluate time from ED visit to arrival at first primary care visit (in days)
     - Unadjusted survival distributions for receiving provider scheduling and transportation versus not and for all 8 intervention arms will be estimated and plotted by the Kaplan-Meier method and compared using the log-rank test.
     - Cox proportional hazards regression model will assess the time from ED visit to arrival at the first primary care visit where all of those who do not have a recorded PCP visit will be censored at the time of last follow-up (last communication/text) or 12 months. The main predictor of interest for these models is assignment to the active primary care provider scheduling and transportation arm (50% of all subjects randomized in the trial), while adjusting for the assignment into other groups (healthy behavior text messages and prompted BP self-monitoring). We will control for age, sex, previous use of antihypertensives, and race. The interactions of these baseline variables will be assessed. The proportionality assumption will be assessed via Schoenfeld residuals by evidence of a non-zero slope in a generalized linear regression of the scaled residuals by time and goodness of fit will be assessed by the Grambsch-Therneau test.

- - To evaluate attendance at two or more primary care visits within 12 months of randomization.
    - This binary endpoint will be assessed using logistic regression. The main predictor of interest for these models is assignment to the active primary care provider scheduling and transportation arm (50% of all subjects randomized in the trial), while adjusting for the assignment into other groups (healthy behavior text messages and prompted BP self-monitoring). We will control for age, sex, previous use of antihypertensives, and race. The interactions of these baseline variables will be assessed. Hosmer-Lemeshow Chi-Squared test will assess the goodness of fit.

1. Since the goal of this exploratory trial is to find interventions and combinations with activity, multiple comparisons will not be controlled.

**2.6 Subgroup analyses**

***Purpose:*** To evaluate the heterogeneity of treatment effects by baseline characteristics of age, sex, previous antihypertensive medication, race, and baseline BP level.

***Methods/Approach:***

- To assess the heterogeneity of treatment effects by age, sex, previous antihypertensive medication and race, the two-way interactions of the main effects of treatments and these covariates will be assessed at the 0.2 significance level
- To assess the heterogeneity of treatment effects by baseline BP, we will divide the cohort into tertiles based on their initial BP level (median of three qualifying SBP from eligibility phase). We will repeat the above primary (Section 2.4) and secondary modeling (Section 2.5a) within each of the three initial BP strata to determine if there is heterogeneity of effect (e.g. do patients with the highest baseline BPs get the most benefit from the intervention components?).

**2.7 Exploratory analyses**

***Endpoints:***

BP

- Proportion of subjects achieving BP control (control = 6/12 month BPs are achieving target recommendations as defined by JNC8)
  - JNC-8 targets for SBP/DBP
  - 2017 AHA guidelines targets for SBP/DBP
- Change in DBP and mean arterial pressure
- Change in 6 month SBP

Process

- Number of interactions (such as calls/texts) to schedule subject initial primary care visit
- Number of interactions to schedule all other primary care visits
- Proportion of BPs texted to the study team
- Proportion enrolled but not randomized

PCP

- Establishment of a primary care providers among those without
- Self-reported number and proportion of provider visits attended
- Proportion of transportation vouchers used

Utilization

- Frequency of Hurley ED and other healthcare visits
- Self-reported follow up of emergency department visits
- Self-reported follow up of provider or clinic visits

Medications

- Changes/escalation in antihypertensive regimen through patient self-report
- Medication Adherence (Hill-Bone Scale, and modified Hill-Bone instrument asking, “How often do you forget to take your HBP medicine?”)

Other

- Social Cognitive Theory (SCT) measures
  - Modified one-question self-efficacy measure, “I am confident that I can take my blood pressure and text it to the Reach Out Team”)
  - Questions querying self-efficacy, motivation, social support and expertise.
    - I am confident that I can control my blood pressure
    - It is worthwhile for me to control my blood pressure
    - My friends and family care if I control my blood pressure
    - I know the right steps to take to control my blood pressure

***Methods/Approach:***

We will similarly assess the impact of the intervention components on the exploratory and process outcomes using linear, Poisson, logistic, or ordinal regression depending on the distributions. The main effects will be tested at the p=0.1 level adjusting for age, sex, previous antihypertensive use, and race. Treatment interactions will be explored at the 0.2 level.

**2.8 Adverse effects**

***Purpose:*** To summarize adverse intervention effects.

***Methods/Approach:*** We will only record adverse events (AEs) and serious adverse events (SAEs) that are possibly, probably, or definitely related to the study interventions.

Adverse effects will be tabulated for each treatment group:

- Number of events by type of event
- Number of subjects by highest grade of event

**3. Post-hoc (data driven) analyses:**

***Purpose:***  (a) To document which analyses were conducted after the results in section 2 were known. (b) To document the rationale for these analyses. (c) To pre-specify their interpretation in the context of the primary and secondary results and their impact on the overall trial conclusions.

***Methods/Approach:***  Specific to the particular setting

**4. Table and Figure Templates:** Outline tables and figures that will be produced for the analysis. This will include all analyses. Tables and figures for the paper will be constructed separately.

- CONSORT diagram of patient flow
- Summary of subject demographics and baseline characteristics overall and by intervention group
- Summary of treatment compliance (snooze, switching from daily to weekly and vice-versa) overall and by intervention group
- Summary of Adverse events by overall and intervention group
- Summary and analysis of change in BP from baseline to Month 12 by main effects of treatments
- Summary and analysis of pattern of change in BP from Baseline to month 12 using all BP measurements
- Summary and analysis of time from ED visit to first PCP visit
- Summary and analysis of proportion of subjects who attend 2 or more PCP visits within 12 months
- Summary and analyses of the exploratory endpoints

1. **Statistical Software**: All analysis will be completed in SAS v9.4 or R

References

1. Moher D, Hopewell S, Schulz KF, Montori V, Gøtzsche PC, Devereaux PJ, Elbourne D, Egger M, Altman DG, Consolidated Standards of Reporting Trials Group. CONSORT 2010 Explanation and Elaboration: Updated guidelines for reporting parallel group randomised trials. J Clin Epidemiol. 2010 Aug; 63(8):e1-37.
2. Altman DG. Comparability of randomised groups. J R Stat Soc. 1985;34:125–136.
3. White, Royston, Wood. 2011. Multiple Imputation using Chained Equations: Issues and guidance for practice. SIM 30: 377-399.
