# Supplementary material for: Reach out behavioral intervention for hypertension initiated in the emergency department connecting multiple health systems: study protocol for a randomized control trial
Source: Trials. 2020 Jun 3;21:456. doi: 10.1186/s13063-020-04340-z (PMC7268693; doi:10.1186/s13063-020-04340-z)
Supplement: Supplementary file 5 — Additional file 5. Recruitment material. [file 13063_2020_4340_MOESM5_ESM.pdf]

REACH OUT is a research project to help you to reduce your high blood pressure. We'll work with you to lower your blood pressure through text messages, send reminders to check your blood pressure, and team up with doctors in the Flint community.

By joining REACH OUT, you will help **build a program that works toward improving how** people manage their blood pressure, in hopes of preventing future health problems such as strokes, heart disease, and kidney disease.

To participate in the REACH OUT project, you will need to…

- ☐ Be 18 years or older
- ☐ Have high blood pressure
- ☐ Have a cell phone that can send and receive text messages
- ☐ Be willing to receive and send text messages (Any text messaging costs will not be paid for by REACH OUT)
- ☐ Take your blood pressure and text it to REACH OUT staff
- ☐ Be willing to come to 2 follow-up visits over the next year – one in 6 months, the other in 12 months.

Will I be paid for my time?

-When you sign up, you will be given a FREE blood pressure cuff and $20.

- o At 3 weeks you will be sent a text message letting you know if you will continue in REACH OUT, or will no longer get text messages

-Those that continue in REACH OUT can earn an additional:

- o $25 at the 6-month follow-up

-And-

- o $30 at the 12-month follow-up

Who should I contact?

For more information, contact Reach Out at reachoutED@med.umich.edu or (810) 337-8399.
