# Supplementary material for: Reach out behavioral intervention for hypertension initiated in the emergency department connecting multiple health systems: study protocol for a randomized control trial
Source: Trials. 2020 Jun 3;21:456. doi: 10.1186/s13063-020-04340-z (PMC7268693; doi:10.1186/s13063-020-04340-z)
Supplement: Supplementary file 6 — Additional file 6. Consent form. [file 13063_2020_4340_MOESM6_ESM.pdf]

UNIVERSITY OF MICHIGAN AND HURLEY MEDICAL CENTER
CONSENT TO BE PART OF A RESEARCH STUDY

1. KEY INFORMATION ABOUT THE RESEARCHERS AND THIS STUDY

Study title: REACH OUT

Company or agency sponsoring the study: National Institute of Health/ National Institute on Minority Health and Health Disparities (NIH/NIMDH)

Names, degrees, and affiliations of the researchers conducting the study:
William Meurer, MD, MS, Department of Emergency Medicine, The University of Michigan
Lesli Skolarus, MD, MS, Department of Neurology, The University of Michigan
Dominic Borgialli, MD, Department of Emergency Medicine, Hurley Medical Center

1.1 <u>Key Study Information</u>

You may be eligible to take part in a research study. This form contains important information that will help you decide whether to join the study. Take the time to carefully review this information. You should talk to the researchers about the study and ask them any questions you have. You may also wish to talk to others such as your family, friends, or other doctors about joining this study. If you decide to join the study, you will be asked to sign this form before you can start study-related activities. Before you do, be sure you understand what the research study is about.

A research study is different from the regular medical care you receive from your doctor. Research studies hope to make discoveries and learn new information about diseases and how to treat them. You should consider the reasons why you might want to join a research study or why it is not the best decision for you at this time.

Research studies do not always offer the possibility of treating your disease or condition. Research studies also have different kinds of risks and risk levels, depending on the type of the study. You may also need to think about other requirements for being in the study. For example, some studies require you to travel to scheduled visits at the study site in Ann Arbor or elsewhere. This may require you to arrange travel, change work schedules, find child care, or make other plans. In your decision to participate in this study, consider all of these matters carefully.
This research is studying whether changing an individual's behaviors may have an impact as a treatment or outcome for high blood pressure (BP). High BP is very common among patients in the Emergency Department (ED). Because of this, we want to test if a text-messaging program could help lower your BP. For the first 3 weeks, you will get text message reminders to take your BP. You will then text the BP result to the study team. If during the 3-week period your blood pressure is still high, we will start the BP program, REACH OUT. REACH OUT is a 12-month text messaging intervention. There are three different types of text messages you could receive while participating in the REACH OUT program (healthy behavior texts, reminders texts to take your BP at home, texts to help with primary care provider appointment scheduling and transportation). This study involves a process called randomization. This means that the combination of text messages, reminders, and appointment assistance you receive in the study is not chosen by you or the researcher. The study design divides study participants into separate groups, based on chance (like the flip of a coin), to compare different treatments or procedures. If you decide to be in the study, you need to be comfortable not knowing which study group you will be in. Any specific instructions and materials for your grouping will be provided to you. If you pay per text message with your cell phone company, regular text messaging costs apply.

IRBMED informed consent template—11-12-2018
Instructions revised 11-12-2018
DO NOT CHANGE THIS FIELD—IRB USE ONLY

APPROVED BY THE
HURLEY MEDICAL CENTER
INSTITUTIONAL REVIEW BOARD
ON November 25, 2019

There can be risks associated with joining any research study. The type of risk may impact whether you decide to join the study. For this study, some of these risks may include asking you about your current recorded BP. The messages are meant to be motivational, but there is a small chance you may be distressed by them. You do not need to answer any question that you don't want to, and you can stop the text messages at any time. There is a small chance that participants in this study could have information about their health status, such as information about their BP levels, or general health activities, given to people they don't want to know. To help prevent this from happening, we will keep any information that might identify you, such as your name and phone number, in a locked filing cabinet or a restricted computer file that is secure and password-protected. We take this risk very seriously and will do our very best to protect your confidentiality, if you decide to participate. There is an extremely small risk that information from the telephone lines used in the automated text messaging system could be intercepted by an outside party. Your phone number will not be given to outside vendors including telemarketers. More detailed information will be provided later in this document.

This study may not offer any benefit to you not, but may benefit others in the future by helping us gain important information about how to help people manage their high BP.

We expect the amount of time you will participate in the study will be about 12 months.

You can decide not to be in this study. Alternatives to joining this study include discussing BP with your primary care physician. The study team can also provide you with informational pamphlets regarding your high BP and how to manage it.

Even if you decide to join the study now, you are free to leave at any time if you change your mind.

More information about this study continues in Section 2 of this document.

2. PURPOSE OF THIS STUDY

2.1 Study purpose:

The purpose of this study is to test whether REACH OUT, a text messaging program, works to lower blood pressure.

3. WHO MAY PARTICIPATE IN THE STUDY

Taking part in this study is completely **voluntary**. You do not have to participate if you don't want to. You may also leave the study at any time. If you leave the study before it is finished, there will be no penalty to you, and you will not lose any benefits to which you are otherwise entitled.

3.1 Who can take part in this study?

You can join this study if you are

- 18 years of age or older
- Found to have high BP while in the ED
- Must have a cell phone with the ability to text-message and willingness to receive texts

3.2 How many people (subjects) are expected to take part in this study?

We expect about 960 participants to be enrolled.

4. INFORMATION ABOUT STUDY PARTICIPATION

4.1 What will happen to me in this study?

APPROVED BY THE
HURLEY MEDICAL CENTER
INSTITUTIONAL REVIEW BOARD
ON November 25, 2019

You will start by completing a survey during your ED visit telling the research team about yourself. We will look at some part of your medical record and medications given in the ED, and throughout the study to see if you have any issues because of REACH OUT.

Once in the study, you will also be given materials about BP. A BP monitor will be given to you to use to take your BP at home. You will be taught how to take your own BP using the monitor given, and how to text these results back to the study team.

On a weekly basis for 3 weeks you will receive text messages reminding you to take your BP. You will then take your own BP and to text the results back to the study team.

If during the 3-week period you still have high BP, the REACH OUT Program will start.

There are three different types of text messages you could receive while participating in the REACH OUT program.

1) healthy behavior texts
2) reminder texts to take your BP at home
3) texts to help with primary care provider appointment scheduling and transportation

It is possible that you might receive all of the types of text messages, or only one type. The type of texts and how often you receive them will depend on what grouping you have been randomly assigned to. You may receive several messages from the study team in one day. It is possible to pause or change frequency of certain types of texts by texting a request to do so. Instructions are provided on the REACH OUT text message instruction sheet.

Follow-up visits will occur at 6 months and 12 months. At these visits, your BP will be measured by the REACH OUT team, and with the home BP cuff given to you at enrollment. You will also be asked to complete additional surveys. The REACH OUT team will meet you at your doctor's office (if you happen to already have a scheduled doctor's appointment around the time of the follow-up visits), in the ED research space, or a mutually convenient location (e.g. home, library, or restaurant).

Throughout the study, and after you are done, we may have a study newsletter that is available to you so you can be updated on the study. At the end of the study period, some participants may be asked to participate in a focus group so we can learn about your opinions on REACH OUT. We may ask you by telephone, or in person to participate. The questions included may be about your health, how you felt about REACH OUT specifically, and in general, and how you felt about the text messages. We may record and will write down notes about what is said during these interviews so that we have a record and can accurately remember your opinions.

This program does not replace your usual health care. If you have a problem or question about your blood pressure or medical problems, you will still need to contact your healthcare team.

As a subject participating in this research study, you have certain responsibilities that may apply to this study, such as ensuring that you arrive at all of your scheduled appointments, take your study medication as directed, and report any adverse reactions you may have during the study.

4.2 How much of my time will be needed to take part in this study?

You will be asked to participate in the study for up to 13 months from when you enroll. You will be asked to complete a survey at the time you enroll in the study, 6 months, and 12 months. These will take about 30-60

APPROVED BY THE
HURLEY MEDICAL CENTER
INSTITUTIONAL REVIEW BOARD
ON November 25, 2019

minutes. You will also receive text messages to text in your BP, which require you to take your BP and text back (about 5 minutes each time). You may also be asked to participate in a focus group or interview about your experience with the program (about 2 hours).

4.3 When will my participation in the study be over?
About 13 months from the day you enroll. It may be slightly longer for those who participate in focus groups.

4.4 What will happen with my information and/or biospecimens used in this study?
Your collected information may be shared with the NIH/ NIMHD.

With appropriate permissions, your collected information may also be shared with other researchers, here, around the world, and with companies.

Your identifiable private information may be stripped of identifiers and used for future research studies or distributed to another researcher for future research studies without additional informed consent.

5. INFORMATION ABOUT RISKS AND BENEFITS

5.1 What risks will I face by taking part in the study? What will the researchers do to protect me against these risks?
The known or expected risks are:
BP monitoring: During the surveys we will ask about your general health, and during the self-monitoring BP text messages we will be asking you about your current recorded BP. The messages are meant to be motivational, but there is a small chance you may be distressed by them. You do not need to answer any question you do not want to, and you can stop the text messages at any time.

Confidentiality: We will collect information about you during the course of the study. There is the possibility that this information could be obtained by non-study staff. We will do everything we can to protect your confidentiality. We will keep any information that might identify you, such as your name and phone number, in a locked filing cabinet or a restricted computer file that is secure and password-protected. We take this risk very seriously and will do our very best to protect your confidentiality, if you decide to participate. Also, there is an extremely small risk that information from the telephone lines used in the automated text messaging system could be intercepted by an outside party. Your phone number will not be given to outside vendors including telemarketers.

Mosio is the company sending out the text messages, and will keep your information confidential and will not share your information with anyone. Mosio may retain a portion of your blood pressures and your phone number throughout the study for quality control purposes and to notify study staff if a problem is later detected with the analysis performed. They will not be used for any reason besides study purposes.

Please note- this program does not take the place of your doctor. Doctors will **not** be evaluating all of the information participants report during their automated text messaging system. If you have any urgent questions, health problems, or dangerous BP levels they should be addressed with your doctor, or call 911.

As with any research study, there may be additional risks that are unknown or unexpected.

5.2 What happens if I get hurt, become sick, or have other problems as a result of this research?
The researchers have taken steps to minimize the risks of this study. Even so, you may still have problems or side effects, even when the researchers are careful to avoid them. Please tell the researchers listed in Section 10 about

APPROVED BY THE
HURLEY MEDICAL CENTER
INSTITUTIONAL REVIEW BOARD
ON November 25, 2019

any injuries, side effects, or other problems that you have during this study. You should also tell your regular doctors.

5.3 If I take part in this study, can I also participate in other studies?
Being in more than one research study at the same time, or even at different times, may increase the risks to you. It may also affect the results of the studies. You should not take part in more than one study without approval from the researchers involved in each study.

5.4 How could I benefit if I take part in this study? How could others benefit?
You may not receive any personal benefits from being in this study. However, we hope that your participation in this study will result in important health benefits to you.

If you agree to participate in the study you may see improvement in your BP. You may learn something new about how to live a healthier lifestyle. Even if you don't benefit personally, the study will help us gain important information about how to help people manage their high BP.

5.5 Will the researchers tell me if they learn of new information that could change my willingness to stay in this study?
Since this is a small, beginning study, we do not anticipate results that will change your care. If you wish to receive a copy of any publication resulting from this study, we are happy to share that with you. You have the option of choosing to possibly receive information about the study (newsletters, results, and other updates) after your participation is over through text, email, or postal mail.

6. OTHER OPTIONS

6.1 If I decide not to take part in this study, what other options do I have?
You do not have to participate in the study, it is completely voluntary. You can discuss managing your BP with your primary care physician. The study team can also provide you with informational pamphlets regarding your high BP and how to manage it.

7. ENDING THE STUDY

7.1 If I want to stop participating in the study, what should I do?
You are free to leave the study at any time. You can follow the instruction on the REACH OUT text message instruction page provided to you at enrollment.

If you leave the study before it is finished, there will be no penalty to you. You will not lose any benefits to which you may otherwise be entitled. If you choose to tell the researchers why you are leaving the study, your reasons for leaving may be kept as part of the study record. If you decide to leave the study before it is finished, please tell one of the persons listed in Section 10 "Contact Information" (below) or follow the instructions on the REACH OUT text message instruction page provided to you at enrollment.

7.2 Could there be any harm to me if I decide to leave the study before it is finished?
We do not know of any harm that may happen if you decided to leave the study before it is finished.

7.3 Could the researchers take me out of the study even if I want to continue to participate?
Yes. There are many reasons why the researchers may need to end your participation in the study. Some examples are:

1. The researcher believes that it is not in your best interest to stay in the study.
2. You become ineligible to participate.

APPROVED BY THE
HURLEY MEDICAL CENTER
INSTITUTIONAL REVIEW BOARD
ON November 25, 2019

3. Your condition changes and you need treatment that is not allowed while you are taking part in the study.
4. You do not follow instructions from the researchers.
5. The study is suspended or canceled.

8. FINANCIAL INFORMATION

8.1 Who will pay for the costs of the study? Will I or my health plan be billed for any costs of the study?
There are no costs or billing for this study. However, if you pay per text message with your cell phone company, regular text messaging costs apply. Additionally, doctor's appointments are not paid for by the study team, and your normal insurance, or coverage process will occur.

You or your health plan will pay for all the things you would have paid for even if you were not in the study, like:

- Health care given during the study as part of your regular care
- Items or services needed to give you study drugs or devices
- Monitoring for side effects or other problems
- Deductibles or co-pays for these items or services.

If you do not have a health plan, or if you think your health plan may not cover these costs during the study, please talk to the researchers listed in Section 10 below or call your health plan's medical reviewer.

By signing this form, you do not give up your right to seek payment if you are harmed as a result of being in this study.

8.2 Will I be paid or given anything for taking part in this study?
You will be given an automated BP cuff and $20 at your enrollment. You will be given $25 after completion of your 6-month follow-up visit, and $30 after completion of your 12-month follow-up visit. REACH OUT will provide transportation, if needed, to the 6 month and 12 month visits.

Some participants may also be provided transportation to their primary doctor for REACH OUT appointments.

Participants asked to participate in the focus groups may receive food and up to $20 following completion of the session.

8.3 Who could profit or financially benefit from the study results?
No person or organization has a financial interest in the outcome of the study.

9. CONFIDENTIALITY OF SUBJECT RECORDS AND AUTHORIZATION TO RELEASE YOUR PROTECTED HEALTH INFORMATION

The information below describes how your privacy and the confidentiality of your research records will be protected in this study.

9.1 How will the researchers protect my privacy?
The known or expected risks are: The risks to participating are minimal. You may experience inconvenience. You may experience some discomfort when taking your blood pressure. This study contains risk associated with transmitting data, including loss of confidentiality.

Taking part in this study will involve collecting private information about you. This information will be protected in the following ways. If there are physical copies of your information, it will be stored in a locked area, accessible only to research staff, and will not be made part of your medical record. Your research information will be entered

APPROVED BY THE
HURLEY MEDICAL CENTER
INSTITUTIONAL REVIEW BOARD
ON November 25, 2019

into a password protected computer and your name will be separated from the data. We will not share data that identifies you with anyone outside of the study. Mosio, a U of M-selected vendor specializing in text message based research and protections will be sending out the text messages throughout the research study. This data will be stored in REDCap. Access to this data is limited to authorized personnel via login to a secure site. The original data on the Mosio server will be deleted to industry standards after study completion. The personal data accessible to Mosio will include your phone number, and the information you text message, including your blood pressure, and potentially information regarding your physician appointments. All study personnel have been trained in how to protect your personal health information and we take this very seriously. The study uses a SSL encrypted web-based application with secure log-ins to access. There is a very small risk that someone could obtain your contact information from the study website.

To help us protect your privacy, we have obtained a Certificate of Confidentiality from the National Institutes of Health. The researchers can use this Certificate to legally refuse to disclose information that may identify you in any federal, state, or local civil, criminal, administrative, legislative, or other proceedings, for example, if there is a court subpoena. The researchers will use the Certificate to resist any demands for information that would identify you. The Certificate cannot be used to resist a demand for information from personnel of the United States federal or state government agency sponsoring the project and that will be used for auditing or program evaluation of agency funded projects or for information that must be disclosed in order to meet the requirements of the federal Food and Drug Administration (FDA). You should understand that a Certificate of Confidentiality does not prevent you or a member of your family from voluntarily releasing information about yourself or your involvement in this research. If an insurer, medical care provider, or other person obtains your written consent to receive research information, then the researchers will not use the Certificate to withhold that information.

A description of this clinical trial will be available on http://www.clinicaltrials.gov/, as required by U.S. Law. This Web site will not include information that can identify you. At most, the Web site will include a summary of the results. You can search this Web site at any time.

9.2 What information about me could be seen by the researchers or by other people? Why? Who might see it?
Signing this form gives the researchers your permission to obtain, use, and share information about you for this study, and is required in order for you to take part in the study.

Medical information and billing records are protected by the privacy regulations of the federal Health Insurance Portability and Accountability Act of 1996 (HIPAA). This type of information is called protected health information (PHI). PHI about you may be obtained from any hospital, doctor, and other health care provider involved in your care, including:

- Hospital/doctor's office records, including test results and dental records
- Any records relating to condition, the treatment received, and response to the treatment
- Demographic information
- Personal identifiers

There are many reasons why information about you may be used or seen by the researchers or others during or after this study. Examples include:

- The researchers may need the information to make sure you can take part in the study.
- University, Food and Drug Administration (FDA), and/or other government officials, auditors, and/or the IRB may need the information to make sure that the study is done in a safe and proper manner.

APPROVED BY THE
HURLEY MEDICAL CENTER
INSTITUTIONAL REVIEW BOARD
ON November 25, 2019

- Study sponsors or funders, or safety monitors or committees, may need the information to:
    - Make sure the study is done safely and properly
    - Learn more about side effects
    - Analyze the results of the study
- The researchers may need to use the information to create a databank of information about your condition or its treatment.
- If you receive any payments for taking part in this study, the University of Michigan accounting department may need your name, address, social security number, payment amount, and related information for tax reporting purposes.
- Federal or State law may require the study team to give information to government agencies. For example, to prevent harm to you or others, or for public health reasons.

The results of this study could be published in an article, but would not include any information that would let others know who you are. We may post a de-identified data set after the study is complete to be shared with other researchers for future research.

9.3 What happens to information about me after the study is over or if I cancel my permission?

As a rule, the researchers will not continue to use or disclose information about you, but will keep it secure until it is destroyed. Sometimes, it may be necessary for information about you to continue to be used or disclosed, even after you have canceled your permission or the study is over.

Examples of reasons for this include:

- To avoid losing study results that have already included your information
- To provide limited information for research, education, or other activities (This information would not include your name, social security number, or anything else that could let others know who you are.)
- To help University and government officials make sure that the study was conducted properly

As long as your information is kept within the University of Michigan Health System, it is protected by the Health System's privacy policies. For more information about these policies, ask for a copy of the University of Michigan "Notice of Privacy Practices". This information is also available on the web at http://www.uofmhealth.org/patient+and+visitor+guide/hipaa. Note that once your information has been shared with others as described under Question 9.2, it may no longer be protected by the privacy regulations of the federal Health Insurance Portability and Accountability Act of 1996 (HIPAA).

9.4 When does my permission expire?

Your permission expires at the end of the study, unless you cancel it sooner. You may cancel your permission at any time by writing to the researchers listed in Section 10 "Contact Information" (below).

10. CONTACT INFORMATION

10.1 Who can I contact about this study?

Please contact the researchers listed below to:

- Obtain more information about the study
- Ask a question about the study procedures or treatments
- Talk about study-related costs to you or your health plan
- Report an illness, injury, or other problem (you may also need to tell your regular doctors)
- Leave the study before it is finished

APPROVED BY THE
HURLEY MEDICAL CENTER
INSTITUTIONAL REVIEW BOARD
ON November 25, 2019

- Express a concern about the study

Principal Investigator: William Meurer
Mailing Address: 1500 E Medical Dr, Ann Arbor, MI
Telephone: 734-615-2766

Study Coordinator: Mackenzie Dome MS
Mailing Address: 1500 E Medical Dr, Ann Arbor, MI

You may also express a concern about a study by contacting the Institutional Review Board listed below.

University of Michigan Medical School Institutional Review Board (IRBMED)
2800 Plymouth Road
Building 520, Room 3214
Ann Arbor, MI 48109-2800
Telephone: 734-763-4768 (For International Studies: US Country Code: 001)
Fax: 734-763-1234
e-mail: irbmed@umich.edu

Hurley Medical Center Institutional Review Board
Research Center
One Hurley Plaza, 6 West, A Wing
Flint, MI 48503
Telephone: 810-257-9974
Fax: 810-262-4768

If you are concerned about a possible violation of your privacy or concerned about a study you may contact the University of Michigan Health System Compliance Help Line at 1-866-990-0111, or Hurley Medical Center Patient Relations office at 1-810-262-9220. *When you call or write about a concern, please provide as much information as possible, including the name of the researcher, the IRBMED number (at the top of this form), and details about the problem. This will help University officials to look into your concern. When reporting a concern, you do not have to give your name unless you want to.*

11. RECORD OF INFORMATION PROVIDED

11.1 What documents will be given to me?

Your signature in the next section means that you have received copies of all of the following documents:

- This "Consent to be Part of a Research Study" document. (*Note: In addition to the copy you receive, copies of this document will be stored in a separate confidential research file and may be entered into your regular Hurley medical record.)*

APPROVED BY THE
HURLEY MEDICAL CENTER
INSTITUTIONAL REVIEW BOARD
ON November 25, 2019

12. SIGNATURES

Consent to Participate in the Research Study

I understand the information printed on this form. I have discussed this study, its risks and potential benefits, and my other choices with ________________________. My questions so far have been answered. I understand that if I have more questions or concerns about the study or my participation as a research subject, I may contact one of the people listed in Section 10 (above). I understand that I will receive a copy of this form at the time I sign it and later upon request. I understand that if my ability to consent or assent for myself changes, either I or my legal representative may be asked to re-consent prior to my continued participation in this study.

Legal Name: ____

Signature: ____

Date of Signature (mm/dd/yy): _________________________

Time (HH:MM): _________________________

- ❑ **I have had the opportunity to ask questions and have those questions answered to my satisfaction.**
- ❑ I would like to receive follow-up newsletters and information regarding Reach Out after completion of the study.
- ❑ If asked to participate in the post-study focus group, I agree to being recorded during discussion.

Principal Investigator or Designee

I have provided this participant and/or his/her legally authorized representative(s) with information about this study that I believe to be accurate and complete. The participant and/or his/her legally authorized representative(s) indicated that he or she understands the nature of the study, including risks and benefits of participating.

Legal Name: ____

Title: ____

Signature: ____

Date of Signature (mm/dd/yy): _________________________

APPROVED BY THE
HURLEY MEDICAL CENTER
INSTITUTIONAL REVIEW BOARD
ON November 25, 2019
